# Snow algae communities in Antarctica: metabolic and taxonomic composition

**DOI:** 10.1111/nph.15701

**Published:** 2019-02-27

**Authors:** Matthew P. Davey, Louisa Norman, Peter Sterk, Maria Huete‐Ortega, Freddy Bunbury, Bradford Kin Wai Loh, Sian Stockton, Lloyd S. Peck, Peter Convey, Kevin K. Newsham, Alison G. Smith

**Affiliations:** ^1^ Department of Plant Sciences University of Cambridge Cambridge CB2 3EA UK; ^2^ Cambridge Institute for Medical Research University of Cambridge Wellcome Trust MRC Building, Hills Road Cambridge CB2 0QQ UK; ^3^ British Antarctic Survey NERC Madingley Road Cambridge CB3 0ET UK

**Keywords:** Antarctica, bacteria, community composition, cryophilic, fungi, metabarcoding, metabolomics, snow algae

## Abstract

- Snow algae are found in snowfields across cold regions of the planet, forming highly visible red and green patches below and on the snow surface. In Antarctica, they contribute significantly to terrestrial net primary productivity due to the paucity of land plants, but our knowledge of these communities is limited. Here we provide the first description of the metabolic and species diversity of green and red snow algae communities from four locations in Ryder Bay (Adelaide Island, 68°S), Antarctic Peninsula.
- During the 2015 austral summer season, we collected samples to measure the metabolic composition of snow algae communities and determined the species composition of these communities using metabarcoding.
- Green communities were protein‐rich, had a high chlorophyll content and contained many metabolites associated with nitrogen and amino acid metabolism. Red communities had a higher carotenoid content and contained more metabolites associated with carbohydrate and fatty acid metabolism. *Chloromonas*,* Chlamydomonas* and *Chlorella* were found in green blooms but only *Chloromonas* was detected in red blooms. Both communities also contained bacteria, protists and fungi.
- These data show the complexity and variation within snow algae communities in Antarctica and provide initial insights into the contribution they make to ecosystem functioning.

## Introduction

Terrestrial life in Antarctica is largely found on the estimated 0.18% of the continent's surface that is ice‐free for at least part of the year (Burton‐Johnson *et al*., 2016; Convey, 2017). But even here, only a small proportion of this exposed area is vegetated. For example, although the Antarctic Peninsula is the most vegetated region of Antarctica, only 1.34% of exposed ground has plant cover (Fretwell *et al*., 2011; Burton‐Johnson *et al*., 2016). However, the actual area of cover by autotrophs may be much higher, as ground‐truthing of satellite imagery has revealed that in many places vegetation comprises not only patches of bryophytes, lichens and higher plants on exposed ground, but also snow algae. Snow algae blooms are often well developed in coastal snowfields as highly visible red and green patches below and on the snow surface where liquid water is present (Fogg, 1967; Broady, 1996; Müller *et al*., 1998). Many snow algal communities consist of either a vegetative stage, seen as green patches in the snow, with *Chloromonas* and *Chlamydomonas* species frequently being the major algal taxa, or an encystment phase (which may also be vegetative), in which the cells have accumulated the keto‐carotenoid astaxanthin, giving rise to red snow patches (Hoham & Duval, 2001; Komárek & Nedbalová, 2007; De Wever *et al*., 2009; Leya, 2013). Fretwell *et al*. (2011) found that areas of snow algae and terrestrial mats in Antarctica could be identified in satellite images in combination with ground‐truthing. If these measurements are typical of terrestrial communities more widely in Antarctica, and considering that a single snow algal ‘bloom’ on the peninsula can cover tens to hundreds of square metres, snow algae are potentially one of the region's most significant photosynthetic primary producers, substantially increasing the known area of land occupied by primary producers in Antarctica. Furthermore, the contribution made by snow algae to terrestrial ecosystem productivity in the Antarctic is likely to be higher than that in the Arctic and other alpine regions, because algal blooms in these other regions tend to be more patchy and occur close to other well‐established and extensive vegetated areas. More widely, these algae play a key role in nutrient dynamics, assimilating nutrients deposited from bird colonies which, as a result of snowmelt, are leached with their associated microbial community into adjacent terrestrial or marine environments, where they support food chains (Dierssen *et al*., 2002; Hodson *et al*., 2008; Boetius *et al*., 2015). Significantly, recent studies of snow algae in the High Arctic have shown that they can alter the albedo of the snow, with darker snow surfaces during red phase algal blooms increasing the local rate of snowmelt (Lutz *et al*., 2016; Cook *et al*., 2017; Ganey *et al*., 2017; Stibal *et al*., 2017).

The Antarctic Peninsula has an extremely variable climate. The region experienced a strong warming period throughout the second half of the 20^th^ century that resulted in increased snowmelt, and at present is undergoing a period of temporary cooling (Turner *et al*., 2009, 2016). Climate warming along the Antarctic Peninsula has resulted in an increase in growing season temperature as well as the availability of water, meaning that two of the major abiotic constraints on biological activity have been relaxed. This may well result in an extended growing season (Vaughan, 2006; Convey, 2011; Chown & Convey, 2012). Thus, there is a potentially large increase in the duration of the algal bloom season associated with a warmer climate in the region. Conversely, cooler periods with a shift in the general wind direction could see the current habitat for snow algae preserved. With habitat regression, or if areas of snow melt completely early in the summer, the ecosystem may be lost entirely for that season (Convey, 2011; Anesio *et al*., 2017). Whichever outcome prevails – which is likely to vary with location – there is an urgent need to study these polar communities to provide a balanced view of polar terrestrial biodiversity and to avoid the loss of these extremophilic primary producers and their community structure at both local and continental scales (Williams *et al*., 2003; Rogers *et al*., 2007; Hamilton & Havig, 2017; Rintoul *et al*., 2018). This is especially pertinent because, although snow algae may not be endemic, there is evidence of endemism and long‐term evolutionary isolation in other associated microbial species and communities around Antarctica, probably due to the geographical isolation of the continent (Vyverman *et al*., 2010; Remias *et al*., 2013; Cavicchioli, 2015; Petz *et al*., 2007).

Despite the ecological importance of Antarctic snow algae, our knowledge of their diversity, distribution, growth and contribution to nutrient cycles is limited to very few locations, such as Goudier Island (64°49′S, 63°29′W) and Paradise Harbour (64°50′S, 62°52′W) (Remias *et al*., 2013). It is currently unknown how prevalent snow algae are in Antarctica, and how much they contribute to primary productivity. Determining the abundance of snow algae will therefore enhance and balance our understanding of the biodiversity of Antarctica. In this study, our objective was to carry out the first estimate of the metabolic and species diversity of snow algae communities collected from four islands in Ryder Bay, adjacent to the Antarctic Peninsula. Specifically, we set out to test whether green and red algae communities have distinct metabolic profiles beyond visual differences in pigmentation. To this end, we investigated the metabolic similarities and differences between green and red blooms in Ryder Bay to identify key shifts in the functional biochemistry of the organisms, the spatial variability of the metabolic composition of snow algae communities and the taxonomic diversity of the communities, in order to identify the algae present and to determine the identity and composition of associated bacterial, protist and fungal communities. To ensure that a wide range of metabolites were detected and identified at this exploratory stage, we used both targeted and untargeted environmental metabolomic approaches in the field (Fourier transform‐infrared spectrometry (FT‐IR)) (to ensure minimal sample degradation) and in the laboratory (HPLC, GC‐flame ionization detection (GC‐FID), GC‐MS) (Bundy *et al*., 2009; Brunetti *et al*., 2013). To assess the quantity of the metabolites in the environment, data were expressed on a per litre of snowmelt basis as well as per unit of dry cell mass. We also used a 16S rRNA gene and internal transcribed spacer (ITS) metabarcoding approach to determine the species composition of the microbial community in the snow algae blooms.

## Materials and Methods

### Field collections in Antarctica

**Figure 1:**
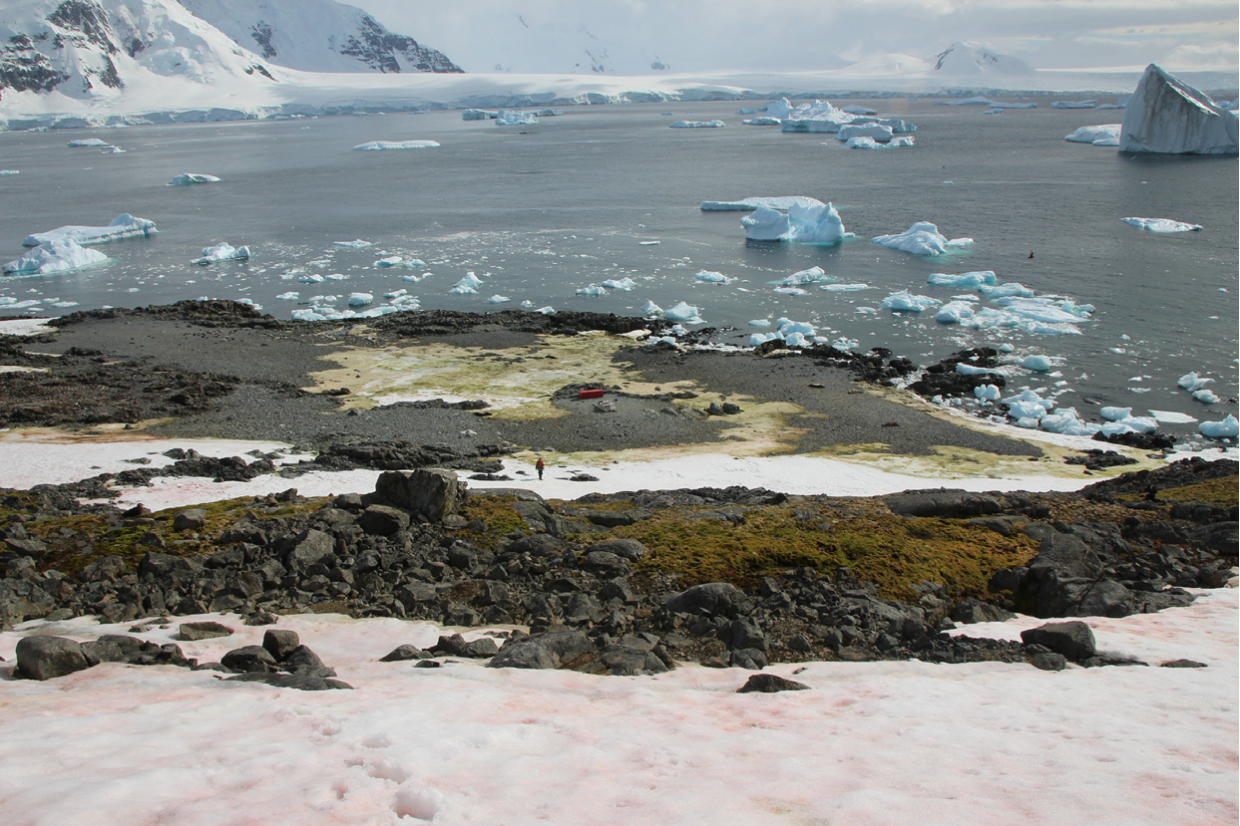
Representative image of snow algal blooms (red‐dominant foreground, green‐dominant midground) in January 2015 on Léonie Island, Ryder Bay, Antarctic Peninsula (see Supporting Information Table S1 for details). Note person in midground for scale.

Snow algae communities (Fig. 1) were collected in 6 × 50 ml sterile plastic sample tubes from layers of green and red dominant snow algal blooms at four locations in Ryder Bay, Antarctic Peninsula (Rothera Point, Anchorage Island, Léonie Island and Lagoon Island) in austral summer (January–February) 2015 (Supporting Information Table**** S1). It was not possible to determine whether all blooms surveyed were successional stages or distinct assemblages. The layers of algae were 1–5 cm deep, but with much heterogeneity within a bloom, with only the algae layer sampled in that 1–5 cm depth. The algae were collected by filling a sterile 50 ml tube with snow, which was not compacted. Seven blooms were studied at Rothera Point (42 samples), nine at Anchorage Island (54 samples), five at Léonie Island (30 samples) and 10 at Lagoon Island (60 samples), resulting in 186 samples for subsequent analyses. Blooms lasted for at least 42 d with bloom areas ranging from *c*. 5 m^2^ to > 2500 m^2^. Single point photosynthetically active radiation (PAR) (Skye PAR Quantum Sensor, Skye Instruments Ltd, Llandrindod Wells, UK) received at the snow surface, together with temperature measurements (standard glass thermometer) at the snow surface and 5 cm depth were also recorded at the start of each sampling period, which lasted for 30–60 min. Samples were returned within 3 h of sampling to the Bonner Laboratory (Rothera Research Station, Ryder Bay, Antarctica), where they were melted in 4°C lit incubators (Sanyo, Osaka, Japan). Algal cell density was measured by adding 6 μl of snowmelt into Hycor Kova haemocytometer wells and counting the number of algal cells using bright field microscopy. Algal community dry cell mass was obtained by gravity filtration of 50 ml of melted snow through a preweighed dry filter (Whatman GF/C, 47 mm). Filters were dried at 80°C for at least 48 h before reweighing. Samples for FT‐IR analysis, which enabled measurements to be obtained as close as possible to the time of sampling ensuring minimal metabolic degradation, were processed by pelleting 2 ml of snowmelt (2000 ***g*** for 10 min at 4°C), discarding the supernatant and drying the pellet at 80°C for 24 h, followed by 24 h in a desiccator. The dried pellets were analysed on station using a Perkin‐Elmer Spectrum Two FT‐IR device, set to measure the absorbance intensity between wavenumbers 400 and 4000 cm^−1^ and normalised against air. For the metabolite and genomic analysis, carried out at the Department of Plant Sciences, Cambridge, 10 ml of snowmelt was pelleted using centrifugation (2000 ***g*** for 10 min, 4°C), after which the supernatant was discarded and the remaining algal pellet was flash frozen in liquid nitrogen and stored at −80°C. Live algae were transported to the UK by adding 20 μl of snowmelt containing algae onto Tris‐acetate‐phosphate (TAP) agar slopes in 30 ml clear plastic tubes for growth under controlled conditions (4°C, 10 μmol m^2^ s^−1^ 12 : 12 h, light : dark cycle) at Cambridge and were imported under UK APHA/DEFRA licence number 119979/260872/0. The slopes and frozen samples were transferred to the UK by ship at 4°C or −80°C.

### Pigment analysis

Total Chl and carotenoid concentrations were determined after extraction of pigments from cell pellets (from 1 ml snowmelt) with 1 ml dimethylformamide using the equations of Inskeep & Bloom (1985) and Wellburn (1994). Individual pigments were analysed by HPLC by first adding 1 ml of deionised water to resuspend the pelleted cells. The resuspended pellets were transferred to a 2 ml microfuge tube and repelleted (16 000 ***g*** for 10 min). The supernatant was removed and the remaining pellet was homogenised with glass beads and frozen in liquid nitrogen three times, after which 1 ml of dimethylformamide was added and sonicated for 30 min. Samples were repelleted and the supernatant was transferred to an HPLC glass vial, mixed with methanol (3 : 2) and stored at −80°C until analysis. Pigments were separated by HPLC (Surveyor system; Thermo Scientific, San Jose, CA, USA) as described by Remias & Lütz (2007) but using an injection volume of 50 μl, and were resolved on a Luna C18 column (250 × 2.0 mm; Phenomenex, Macclesfield, UK). Peaks were compared against standards (astaxanthin and astaxanthin esters, lutein, Chl*a* and Chl*b*, and β‐carotene; all Sigma Aldrich) (Inskeep & Bloom, 1985; Wellburn, 1994).

### Total cellular lipids and FAMEs

Lipids were extracted using the chloroform/methanol/water method and triacylglycerides (TAGs), polar lipids and free fatty acids in the total lipid extract and total fatty acid methyl esters (FAMEs) were analysed by GC, as described in Davey *et al*. (2014).

### Metabolite profiling

Soluble polar and nonpolar metabolites were extracted using the methanol/chloroform/water method as described in Davey *et al*. (2008). Compounds within the polar methanol/water phase were derivatised by *N*‐methyl‐*N*‐trimethylsilyl‐trifluoroacetamide (MSTFA) and trimethylsilyl (TMS) as described by Dunn *et al*. (2011) and subsequently separated and profiled by GC‐MS (Thermo Scientific Trace 1310 GC with ISQ LT MS, xcaliber v.2.2) with a ZB‐5MSi column (30 m, 0.25 mm ID, 0.25 μm film thickness; Phenomenex). The injection volume was 1 μl (splitless) with an injector temperature of 300°C, using helium as a carrier gas (constant flow rate of 1.0 ml min^−1^). The following gradient was used: initial oven temperature 70°C; 130°C at 10°C min^−1^; 230°C at 5°C min^−1^; 310°C at 20°C min^−1^; hold for 5 min. The MS conditions in the positive mode were: transfer line 310°C; ion source, 310°C; mass range 45–800 Da; dwell time 0.17 amu s^−1^. GC‐MS spectra were aligned to an internal standard (phenyl‐β‐d‐glucopyranoside hydrate 98%, Davey *et al*., 2008) and processed using thermo tracefinder (v.3.1) and nist software (nist v.2.0 http://www.nist.gov/srd/nist1a.cfm) to aid identification based on molecular mass. Pathway analysis of the identified metabolites used metaboanalyst open source software (v.4, pathway analysis tool) using the *Arabidopsis thaliana* metabolic pathway library (Chong *et al*., 2018, www.metaboanalyst.ca). R script for the metaboanalyst software can be downloaded at https://github.com/xia-lab/MetaboAnalystR (Chong & Xia, 2018).

### Metabarcoding

Frozen pellets (*c*. 1 cm^3^) of field‐collected algal communities from 10 ml snowmelt were allowed to thaw before being resuspended in 1 ml of RNase‐free water. After transferring to a clean 1.5 ml microfuge tube, the samples were ground with sterilised sand before adding another 1 ml of RNase‐free water and subsequent transfer to a 15 ml tube to which 3 ml of SDS‐EB buffer (2% SDS, 400 mM NaCl, 40 mM EDTA, 100 mM Tris‐HCl, pH 8.0) was added, followed by mixing by vortexing and shaking for 5 min at 4°C. Subsequently, 3 ml of chloroform was added, mixed gently by inversion and the whole suspension was centrifuged for 5 min at 2000 ***g*** and 4°C, resulting in a two‐phase separation. The top aqueous phase was transferred to a new 15 ml tube and two volumes of 100% chilled ethanol were added before incubating overnight at −20°C. The following day, the mix was spun at 6800 ***g*** at 0°C for 30 min. After carefully discharging the supernatant, the pellet was resuspended with 1 ml of ethanol (70%) and recovered in a clean microfuge tube before determining total RNA concentration and quality. Libraries of the fourth hypervariable (V4) domain of the 16S rRNA gene and ITS of the rRNA gene were produced using the NEXTflex ‘16S V4’ and ‘18S ITS’ Amplicon‐Seq Library Prep Kit and primers (BIOO Scientific, Austin, TX, USA), respectively. For consistency we hereafter use the term ‘ITS’ for the NEXTflex 18S ITS region. The microbial 16S rRNA gene forward primer (V4 Forward) sequence was: 5′‐GACGCTCTTCCGATCTTATGGTAATTGTGTGCCAGCMGCCGCGGTAA‐3′ and the reverse primer (V4 Reverse) sequence was: 5′‐TGTGCTCTTCCGATCTAGTCAGTCAGCCGGACTACHVGGGTWTCTAAT‐3′. The eukaryotic ITS forward primer (18S ITS Forward) sequence was: 5′‐CTCTTTCCCTACACGACGCTCTTCCGATCTTCCGTAGGTGAACCTGCGG‐3′ and the reverse primer (18S ITS Forward) was 5′‐ CTGGAGTTCAGACGTGTGCTCTTCCGATCTTCCTCCGCTTATTGATATGC‐3′. Samples were sequenced by Cambridge Genomic Services (Cambridge, UK) using an Illumina MiSeq v3 600‐Cycle Sequencer following the manufacturer's protocol and primers. Quality control analysis of the Illumina MiSeq paired‐end reads (2 × 300 bp) was performed using fastqc (https://www.bioinformatics.babraham.ac.uk/projects/fastqc/). Taxonomic analysis of 16S rRNA gene sequences was performed using qiime 2 release 2017.10 (Caporaso *et al*., 2010; https://qiime2.org). In brief, for each sample, demultiplexed paired‐end sequences were imported into qiime 2. Potential amplicon sequence errors were corrected with the qiime 2 implementation of dada2 (Callahan *et al*., 2016). To remove lower quality bases, reads were truncated at position 280 based on the fastqc reports during this step. Taxonomy was assigned to the sequences in the feature table generated by dada2 using silva release 128 as the 16S/ITS(18S) marker gene reference database (Quast *et al*., 2013), trimmed to the V4 region, bound by the 515F/806R primer pair used for amplification. Taxonomic analysis of the ITS sequence data was done as described for the 16S rRNA gene up to the taxonomic assignment step. Because of the lack of an ITS marker reference database representative of the species diversity of the environments investigated, we carried out sequence similarity searches using NCBI blast (Altschul *et al*., 1990) against release 134 of the European Nucleotide Archive (ENA). Taxonomic assignments were made manually, based on blast scores and the presence or absence of ambiguity of the taxonomic lineages reported by blast. For ambiguous blast hits (e.g. a similar score for unrelated taxa), the lowest common denominator was used for taxonomic assignment. Since there were so few operational taxonomic units (OTUs), and in the absence of an ITS database that is representative of the communities under investigation, we treated these results as exploratory. Sequence reads were submitted to the ENA Sequence Read Archive at the European Bioinformatics Institute (https://www.ebi.ac.uk/ena) and are available under accession number PRJEB23732.

### Targeted 18S rRNA gene PCR for isolated snow algae species

Snow algae were isolated from a field sample (Lagoon Island) and grown axenically on TAP agar plates supplemented with ampicillin (50 μg ml^−**1**^) and kasugamycin (50 μg ml^−1^). Cultures were maintained under a 12 : 12, light : dark photoperiod at 4°C. PCR mixtures contained REDTaq ReadyMix PCR Reaction Mix (Sigma) with 10 μM of the 18S rRNA gene universal eukaryotic primers (forward sequence ‘SA Forward’: 5′‐CGGTAATYCCAGCTCCAATAGC‐3′, reverse sequence ‘SA Reverse’: 5′‐GTGCCCTTCCGTCAATTCC‐3′), with expected product size of 582–584 bp. Primers were adapted from Wang *et al*. (2014) where our forward primer was a 3′ section of the first primer in their Table 1, with the addition of nucleotides downstream of it so the primers would have similar annealing temperatures, avoiding a run of guanines and cytosines and to avoid the possibility of forming stable secondary structures or primer dimers. Our reverse primer is the reverse complement of a section of the second listed primer in their Table 1. The PCR cycle was 95°C for 5 min, 95°C for 20 s, 55°C for 20 s, 72°C for 1 min (35 cycles), and 68°C for 5 min. PCR products were extracted from the gel using a QIAquick Gel Extraction kit (Qiagen), following the manufacturer's instructions, and nucleotide sequencing (both directions using the above primers) was performed using Applied Biosystem sequencing platforms (Abi 3730xl genome analyser, 50 cm 96 capillary array) at Source BioScience (Cambridge, UK) and viewed using snapgene v.4.2.4. Nucleotide sequences were deposited at GenBank and are available under accession numbers MK330877–MK330880.

**Table 1:** Pigment composition of snow algae

Pigment		Green community (mg g^−1^ DCM)	Red community (mg g^−1^ DCM)
**Chl** ***a***	***↓***	3.37 (1.24)	0.28 (0.11)*
**Chl** ***b***	***↓***	1.54 (0.47)	0.13 (0.06)*
Chl‐like	***↓***	1.59 (0.66)	0.00 (0.00)*
**β‐Carotene**	***↓***	0.40 (0.16)	0.00 (0.00)*
β‐Carotene‐like	***↓***	0.02 (0.02)	0.00 (0.00)
**Lutein**	***↓***	0.58 (0.19)	0.01 (0.01)*
Xanthophyll	***↓***	0.21 (0.07)	0.06 (0.03)
Astaxanthin‐like	***↑***	0.03 (0.02)	0.07 (0.02)
**Astaxanthin esters**	***↑***	0.34 (0.13)	0.63 (0.30)

Pigments are expressed as mg g^−1^ dry cell mass (DCM) from green and red snow algal communities collected from four locations in the maritime Antarctic (Rothera Point, Anchorage Island, Léonie Island and Lagoon Island) during austral summer (January–February) 2015. Data were pooled from all collection sites (mean ± SE, *n *=* *6). Dominant pigments are highlighted in bold.

* *P* ≤ 0.05 between green and red communities. Arrows show the trend of change from green to red communities.

### Statistics

To determine whether the differences between green and red communities, or among bloom site locations, were statistically significant, *t*‐tests (Excel, Microsoft Office 2007) or two‐way ANOVA with Tukey's test (sigmaplot v.13.0, Systat Software Inc., Chicago, IL, USA) were performed. Multivariate analyses to test whether green and red communities could be discriminated based on their identified and unidentified metabolites (from FT‐IR fingerprints or GC‐MS profiling datasets) were performed using principal components analysis (PCA) (Paliy & Shankar, 2016) on unit‐variance scaled data (FT‐IR absorbance values or GC‐MS identified peak area units) within the simca‐P v14.1 PCA pipeline (Umetrics, Umeå, Sweden) to produce standard score scatter plots and ranked score contribution plots of how each variable (FT‐IR wavenumber or GC‐MS metabolite) contributed to clustering within the PCA score scatter plot.

Alpha diversity and beta diversity were measured using qiime 2's diversity analyses (q2‐diversity) plugin (version 2017.10; https://docs.qiime2.org/2017.10/tutorials/moving-pictures/). In brief, the core‐metrics‐phylogenetic method was applied, which first subsamples the counts from each sample in order to obtain an even sampling depth (43 828 for the 16S rRNA data and 69 for the ITS data). Alpha and beta diversity metrics (Faith's Phylogenetic Diversity and Pielou's Evenness) were subsequently computed (PERMANOVA). To determine whether the richness of the samples had been fully captured, alpha diversity rarefaction plots were calculated using the qiime diversity alpha‐rarefaction visualiser. Plots for 16S rRNA gene and ITS data approached saturation at depths of 43 828 and 69 sequences, respectively (Fig.**** S1), suggesting that the majority of the diversity in the communities had been captured. Beta diversity nonmetric multidimensional scaling (NDMS) plot analysis of the raw read metabarcoding data was carried out using r script in vegan (Methods**** S1; https://jonlefcheck.net/2012/10/24/nmds-tutorial-in-r/) with stress levels of 0.1–0.2, using the Bray–Curtis dissimilarity calculation (Paliy & Shankar, 2016). Given the limited access to some blooms, and to reduce the environmental impact, the sample numbers (*n*) varied (1–30) per site, and these sample numbers are given in the figure and table legends.

## Results

### Algal community cell density and biomass

There were many fewer cells in the red algal communities than in the green communities, with mean (± SD) cell densities of 0.15 × 10^6^ cells per ml snowmelt (± 0.17 × 10^6^) vs 1.24 × 10^6^ cells per ml snowmelt (± 0.79 × 10^6^), respectively (Fig.**** S2a). Similarly, the two communities differed in dry biomass (mg l^−1^ of snowmelt), with, on average across all sites, 62% less biomass in the red‐dominated communities than in the green communities (Fig**.** S2b). There were no significant differences (ANOVA, *P *>* *0.05) in cell numbers or cell dry masses between sampling locations in the red communities. However, the green communities on Léonie Island had a greater number of cells (*c. *2.2 × 10^6^ more cells per ml) and biomass (*c. *2.9 g dry mass more per litre) of snowmelt than on the other islands (ANOVA, *P* ≤ 0.01).

### FT‐IR metabolite fingerprinting

**Figure 2:**
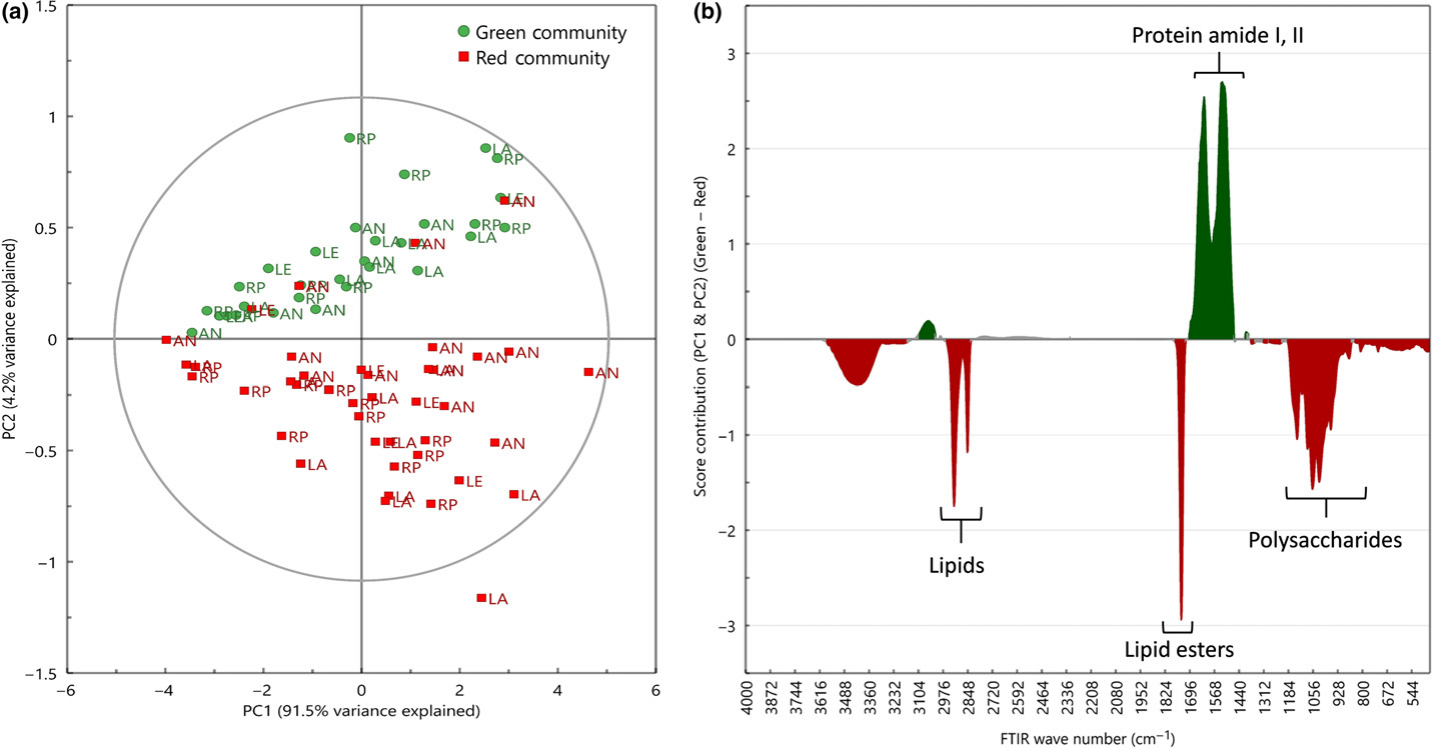
Metabolic fingerprinting (FT‐IR) reveals differences between green and red blooms. Score scatter plot (a) from principal component analysis of FT‐IR wavenumber intensities of green (circles, *n *=* *30) and red (squares, *n *=* *40) snow algae communities collected from four locations adjacent to the Antarctic Peninsula (Rothera Point (RP), Anchorage Island (AN), Léonie Island (LE) and Lagoon Island (LA)) during January and February 2015 (austral summer). The score contribution plot (b) indicates which FT‐IR wavenumbers differ the most between green (protein, amide I, II) and red (lipids, lipid esters, polysaccharides) snow algae communities along PC1 and PC2.

FT‐IR was used during the field campaign to analyse the metabolic composition of the snow algae communities as close to field conditions as possible (Fig. 2a). Based on PCA, there were specific FT‐IR wavenumber regions that were strongly associated with green (1489–1581, 1589–1664 cm^−1^) or red (1002–1094, 1141–1144, 1732–1756, 2850–2856, 2911–2933 cm^−1^) snow algae communities (Fig. 2b). These regions were associated with protein bands (amide I and II) – suggesting active growth – in the green communities, whereas in red communities the major features were associated with lipids, lipid esters and polysaccharides (Fig. 2b; Table**** S2). There was no clustering of samples based on sample island location. Little variation across island locations suggests that the sampling regime was standardised and robust.

### Pigments composition in snow algae blooms

Crude solvent extractions and ultraviolet–visible spectrometry of green and red blooms shortly after field collection showed the presence of peaks indicative of Chl*a* and *b* and astaxanthin (Fig.**** S3). There were significant differences in the total Chl content of green‐ and red‐dominant snow algae communities, with more total Chl present in the green than in the red communities when expressed on a per unit dry mass (*c. *83% more) and per volume snowmelt (*c. *90% more) (Fig.**** S4a,b). There was no difference in the concentrations of total carotenoid between the red and green blooms, when expressed either on a per unit dry mass basis (Fig.**** S4c) or on a per litre of snowmelt (Fig.**** S4d) basis. There was a single effect of location, with the Chl content of the Léonie Island green community having more Chl per unit of snowmelt than the other islands (*P* ≤ 0.05), which was probably due to the greater biomass per unit of snowmelt at that location. There were no other effects of location on total Chl or total carotenoid in the red communities.

Pigment composition was analysed in detail using HPLC. As there was minimal effect of location from the above pigment analyses, samples from the islands were pooled to provide an average composition over Ryder Bay for green and red communities (Table 1). Dominant pigments in the green community were Chl*a* and *b*, β‐carotene and lutein, and in the red community were Chl*a* and *b* and astaxanthin esters. The main differences between the communities were that the red communities had significantly less Chl*a* and *b*, β‐carotene, lutein (*t*‐test, *P* ≤ 0.05) and xanthophyll and more astaxanthin‐like and astaxanthin esters, although these were not significant (Table**** 1). The ratio of Chl*a* to Chl*b* was similar in both green (1.9 : 1) and red (2.2 : 1) communities.

### Lipid profiling of green and red blooms

**Figure 3:**
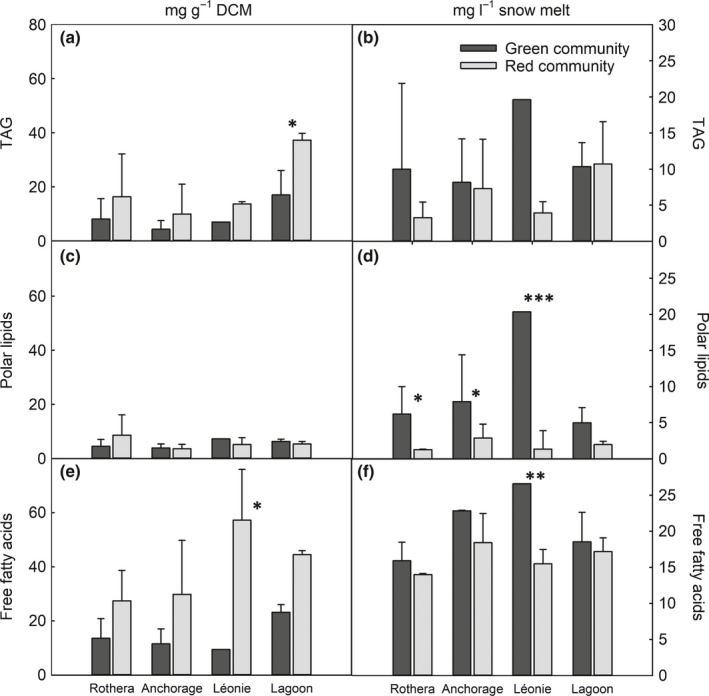
Lipid content of snow algae blooms. Total triacylglycerides (TAGs), membrane lipids and free fatty acids (as C16 equivalent) are expressed as both mg g^−1^ dry cell mass (DCM; a, c, e) and mg l^−1^ snowmelt (b, d, f) from green and red snow algae communities collected from four locations adjacent to the Antarctic Peninsula (Rothera Point, Anchorage Island, Léonie Island and Lagoon Island) during January and February 2015 (austral summer). Data are mean ± SD. Total green and red sample sizes (*n*) are: RP 4, 3; AN 3, 7; LE 1, 2; LG 3, 3. Statistical differences (ANOVA) between green and red communities within a location are denoted by: *, *P* ≤ 0.05; **, *P* ≤ 0.01; ***, *P* ≤ 0.001.

More detailed analyses using GC‐FID and GC‐MS were performed to qualify and quantify differences between communities identified from FT‐IR spectra. We first measured the overall glycerolipid composition of the samples (expressed as mg per unit dry cell mass). Unlike the other metabolites detected, there was little variation in the nonpolar (TAG) and polar lipid content between the green and red communities, nor between the different island locations. The exception to this was significantly (*P* ≤ 0.05) more neutral storage lipids (TAGs) per unit dry mass in the lipid extracts from the red community at Lagoon Island (Fig. 3a). A significantly higher concentration of free fatty acids was also measured in the red communities from Léonie Island (Fig. 3e). However, when data were expressed per litre of snowmelt, the polar membrane lipid content of the red community from Léonie Island was less than that of the green community (*P* ≤ 0.01; Fig. 3d), and the mean free fatty acid concentration in the red communities was lower compared to the green communities from all locations except Lagoon Island (*P* ≤ 0.05; Fig. 3f). There were no significant differences in lipid composition in either the red or the green communities between the sampling locations.

FAME analyses showed that the snow algae communities contained a range of saturated and unsaturated fatty acids from C14:0 to C22:6 and were rich in the saturated C16:0 and unsaturated C18:1(11) fatty acids (Table**** S3). Overall, there were no statistically significant differences in the fatty acid profile between the green and red communities, but the trend was for greater amounts of C16:0, C18:1(11) and C18:1(9) fatty acids in the red community, and lower or similar amounts of all other fatty acids.

### Metabolic profiles of red and green snow algae communities

**Figure 4:**
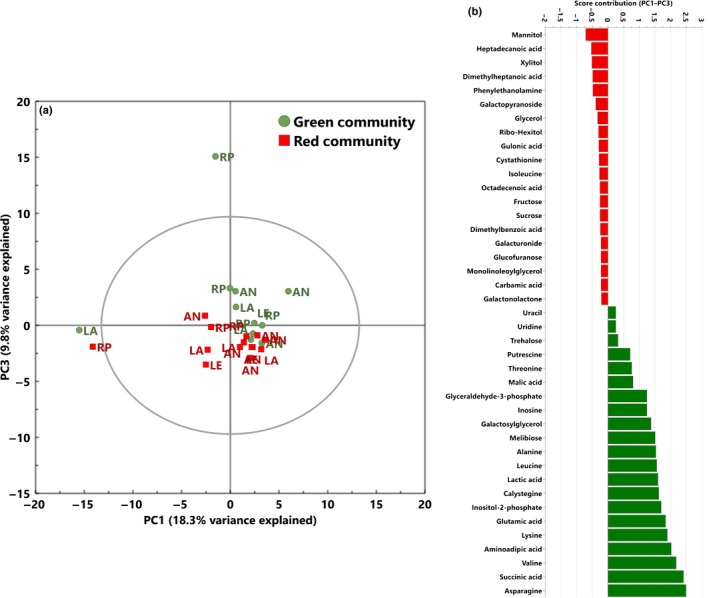
Metabolic profiling (GC‐MS) reveals differences between green and red blooms. Score scatter plot (a) from principal component analysis (PCA) of putatively identified metabolite intensities (GC‐MS) of green (circles, *n *=* *11) and red (squares, *n *=* *14) snow algae communities collected from four locations adjacent to the Antarctic Peninsula (Rothera Point (RP), Anchorage Island (AN), Léonie Island (LE) and Lagoon Island (LA)) during January and February 2015 (austral summer). The score contribution plot (b) values (top 20) are ranked in order of importance and are positive if they contribute towards PCA loading plots for the green snow algae communities and negative if they contribute towards the red snow algae communities. The full list of metabolites is presented in Supporting Information Table S4.

To provide further insight into the metabolic composition of the different snow algae communities, an untargeted metabolic profiling approach was used in which the extracts were derivatised by MSTFA and analysed by GC‐MS, and the peaks identified. PCAs showed distinct clustering of green and red communities (Fig. 4a). There was no clustering of samples based on island location in any of the principal components. The score contribution of metabolites (based on their molecular masses and comparisons with NIST MS libraries) and the metabolic pathways in which they are involved were ranked in order of importance for either green or red snow algae communities using the *in silico* MetaboAnalyst Pathway Analysis Tool. Metabolites involved in energy production and the TCA cycle and in nitrogen and amino acid metabolism, such as succinic acid and the amino acids asparagine and valine, were strongly associated with green snow algae communities (Fig. 4b; Tables**** S4, S5). The most frequent metabolites associated with red communities were quite different from those in the green communities. They were largely associated with osmolytes (mannitol, xylitol) and the fatty acids heptadeconoic acid (an unsaturated C17 fatty acid) and dimethyl‐heptanoic acid (a C7 volatile acid).

### Snow algae community composition

Bright‐field microscopy revealed mainly flagellated and nonflagellated green algal cells representing the vegetative stage in green blooms, and orange to red mature zygospores or large hypnozygotes in red blooms (Fig.**** S3). Although green algal blooms in Ryder Bay were observed to become red over periods of about 30 d, given the remote location, which precluded many repeat samplings, it could not be irrefutably established whether the red forms of the cells were derived from the green vegetative cell forms, or if the red cells were a separate assembly that succeeded green blooms.

We therefore carried out metabarcoding analysis to investigate the species composition of the communities, via sequencing libraries of the V4 region of 16S rRNA gene and ITS region of each community and then by NMDS plot analysis of the 16S rRNA gene or ITS OTUs. Both read frequencies and percentage contributions were obtained for two major taxonomic levels (Kingdom/Phylum Level 2 to Genus Level 7). To test for associations between discrete metadata categories (green and red algae communities) and alpha diversity data, the community richness and evenness were calculated using Faith's Phylogenetic Diversity (a qualitative measure of community richness that incorporates phylogenetic relationships between the features) and Pielou's Evenness (a measure of community evenness). No significant differences in community richness or evenness were measured between green and red 16S rRNA gene and ITS sequence‐based communities (*P* ≥ 0.05, Kruskal–Wallis test) (Fig.**** S5). For beta diversity, a PERMANOVA (Anderson, 2001) test (using qiime 2's beta‐group‐significance command) on unweighted UniFrac distances generated during the first diversity analysis step was used to test whether sequence reads from samples within a bloom type were more similar to each other than they were to samples from the other bloom type. Similar to alpha diversity, there was no significant dissimilarity between the green and red 16S rRNA gene and ITS communities (*P* ≥ 0.05) (Fig**.** S6). Beta diversity NMDS plots of both 16S rRNA gene and ITS data sets also revealed close taxonomic similarities between the green and red communities (Fig.**** S7).

**Figure 5:**
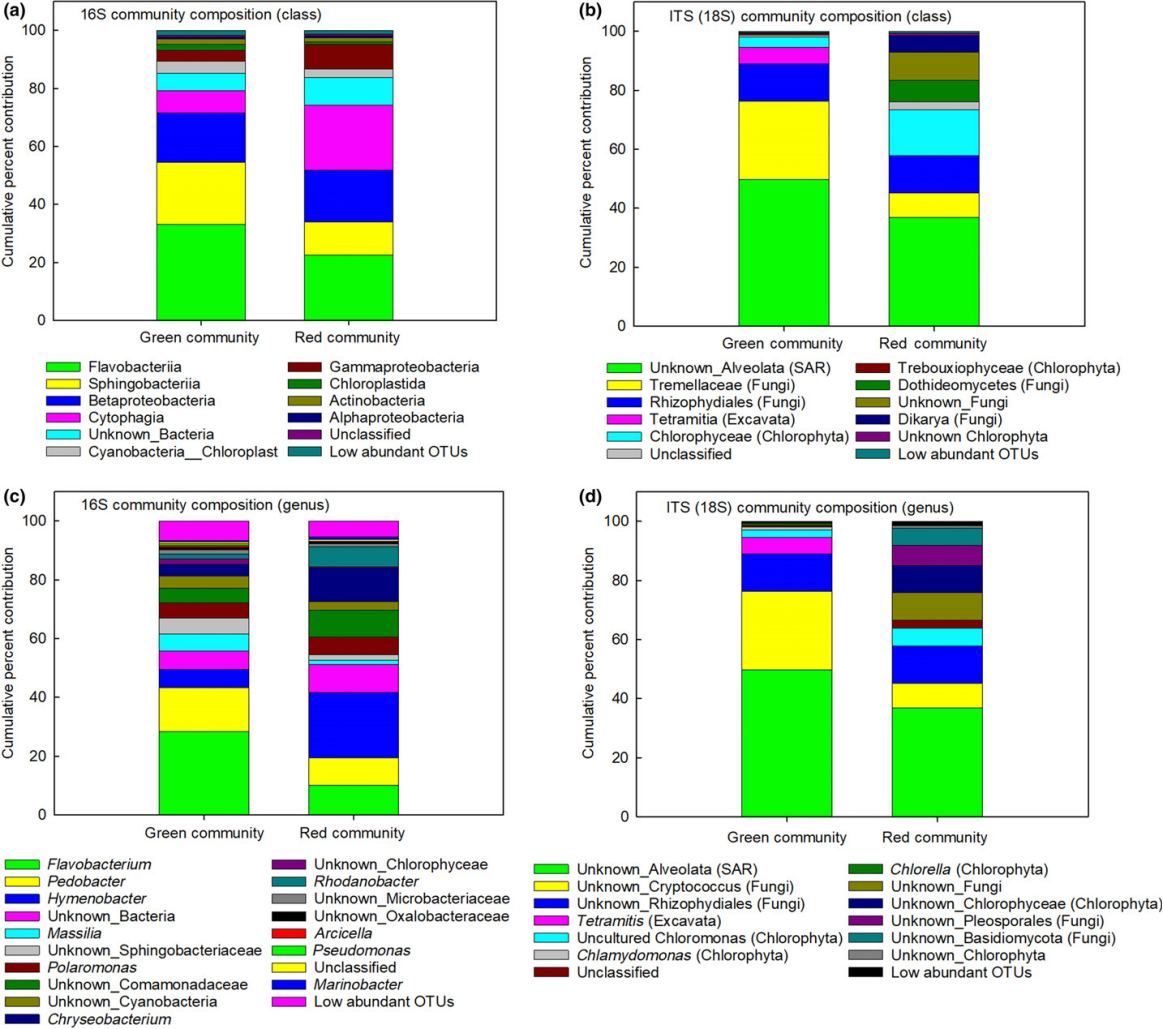
Taxonomic composition of snow algae blooms. Per cent contribution of taxonomic assignments for 99% aligned operational taxonomic units (OTUs) for 16S rRNA and 18S ITS1 sequences in green and red snow algae communities from Ryder Bay, Antarctica, during January and February 2015 (austral summer). Per cent contribution values are the mean relative abundance of the taxa as a percentage of total sequences with < 0.5% abundance and are classified at the (a, b) class or (c, d) genus level. Low abundance OTU values are the sum of the percentages for taxa identified below 0.5% contribution. All values are the mean of *n *=* *5 (green community sites) or *n *=* *6 (red community sites). SAR, ‘stramenopiles, alveolata, rhizaria’. Detailed OTU read numbers, per cent contributions and statistics are presented in Supporting Information Tables S6–S8.

Despite being the major observable organisms in the samples, the Chlorophyta contribute little to the overall diversity, which was instead dominated by fungi, other protists and bacteria (Fig. 5; Tables**** S6–S8). However, of note is the difference in the Chlorophyta between the green and red blooms: in the green blooms OTUs whose closest hit in the databases were to *Chloromonas*,* Chlamydomonas* and *Chlorella* were detected in approximately equal measures, whereas in the red blooms only *Chloromonas* was identified, with the other OTUs being assigned to unknown Chlorophytes (Fig.**** S8), indicating that the red community contains other, unidentified green algal species. Further investigation into the identity of the snow algae in the red blooms, based on preliminary analysis of their morphology, suggested that they could either be *Chlamydomonas nivalis* or *Chloromonas nivalis*. 18S rRNA gene PCR was thus performed on nucleotide extracts from red snow algae cultures that were isolated from a field sample (Lagoon Island) and grown axenically, during which the cells transformed from their red phase to a green phase (over 21 d). A blast search of the forward and reverse nucleotide sequences resulted in 98–99% similarity to *Chloromonas* sp. and *Chlamydomonas* sp., supporting our initial classification. However, a blast search against only *Chlamydomonas nivalis* or *Chloromonas nivalis* sequences resulted in just 92% similarity (Table**** S9), indicating that these might be other species. At the class level, sequence reads from the 16S rRNA gene metabarcoding showed that the communities were dominated by *Flavobacteria*,* Sphingobacteria* and beta‐proteobacteria, in particular *Flavobacterium*,* Pedobacter* and *Hymenobacter*, respectively (Fig. 5a**)**. The number of Sphingobacteria OTUs was statistically significantly lower (*P *<* *0.05) and *Chryseobacterium* reads significantly higher (*P *<* *0.01) in the red communities compared to the green communities (Tables**** S7, S8).

## Discussion

Our objective was to carry out the first estimate of the metabolic and species diversity of snow algae communities collected from four islands in Ryder Bay, adjacent to the Antarctic Peninsula. Our study demonstrates that green and red Antarctic snow algae communities have unique biochemical profiles beyond the observable differences in pigmentation and are members of complex microbial communities that include a range of bacterial, protist and fungal taxa.

### Metabolic composition differs between green and red snow algae blooms

From direct field analyses, we have shown that there are substantial differences in biomass and cell densities between green and red blooms. Additionally, the initial FT‐IR untargeted metabolic profiling revealed that wavenumbers associated with protein/amino acids were more abundant in the green blooms, whereas lipid and carbohydrate chemistry predominated in the red blooms. Such differences in FT‐IR spectra between the green and red communities were similar to those detected by single‐celled synchrotron‐based IR spectroscopy of Arctic snow algae communities by Lutz *et al*. (2015), who found that green communities were dominated by functional groups associated with proteins and red communities by lipids.

### Astaxanthin increases and Chl content decreases in red blooms

Snow surface pigmentation is a fundamental marker for identifying and classifying snow algae communities during field campaigns and in research based on satellite images (Fretwell *et al*., 2011). However, it cannot be assumed that pigment composition does not vary between blooms, especially as the species composition of the green and red communities have not been previously described. We found that between green and red communities, although the composition of the pigments was similar, the concentrations of each pigment was not. Our pigment data for the Ryder Bay snow algal communities were consistent with those of other snow algal blooms around the world. For example, Remias *et al*. (2010, 2013) and Lutz *et al*. (2015, 2016, 2017) reported higher concentrations of Chl and xanthophyll cycle‐related compounds in green blooms, but, unlike Remias *et al*. (2010), we were unable to detect α‐tocopherol (vitamin E) in the cells. The detected carotenoids potentially play a key role in energy dissipation in chloroplasts under high light conditions (Demmig‐Adams & Adams, 1996; Remias *et al*., 2010). Higher concentrations of astaxanthin esters in red blooms have also been reported elsewhere (Remias & Lütz, 2007; Lutz *et al*., 2015), the production of which can be dependent on developmental stage (Holzinger *et al*., 2016) or upon environmental stresses, in particular light intensity and nutrient deficiency (Remias *et al*., 2005, 2010; Lutz *et al*., 2014; Minhas *et al*., 2016).

### Concentrations of free fatty acids vary, but those of glycerolipids largely do not, between green and red blooms

Our analyses with both untargeted and targeted metabolomic profiling approaches (Bundy *et al*., 2009) reveal many differences and similarities between the green and red bloom communities and confirmed the field findings from the FT‐IR data. Lipid profiling showed that only the free fatty acid concentrations differed between bloom types (with higher concentrations in the red communities), and that the glycerolipid and fatty acid composition was similar between blooms. Such profiles are characteristic of other snow algae blooms, in which the high degree of fatty acid saturation is hypothesised to be related to membrane stability at low temperatures (Bidigare & Ondrusek, 1993; Spijkerman *et al*., 2012; Leya, 2013). Specifically, this is characteristic of *Chlamydomonas nivalis* (Řezanka *et al*., 2014), with Lukeš *et al*. (2014) relating the membrane lipid composition to a broad thermal tolerance in terms of growth, electron transport and oxygen evolution, compared to the temperate species *Chlamydomonas reinhardtii*.

### Distinct metabolic profiles of red and green snow algae communities

GC‐MS profiling revealed that the dominant metabolites and metabolic pathways in green blooms were associated with nitrogen and amino acid metabolism, and in red blooms with osmolyte and fatty acid metabolism. High on the list of compounds associated with green communities were lysine and its precursor aminoadipic acid, the latter importantly being a precursor for penicillin synthesis in fungi that produce α‐aminoadipate (Fazius *et al*., 2012). Also dominant in the green blooms was calystegine, an alkaloid involved in plant–bacterial communication, including with *Pseudomonas* (a reported bacterial genus in our 16S sequencing), which is reported to catabolise it (Goldmann *et al*., 1996). Glycerol, sugar alcohols and other low‐molecular‐weight carbohydrates have been reported previously in red snow algae, with their function associated with osmotic acclimation (Eggert & Karsten, 2010). Remias *et al*. (2013) also detected high concentrations of glycerol and sugar alcohols in red blooms in Antarctica. By contrast, studies of snow algal communities in the High Arctic by Lutz *et al*. (2015) found that compounds related to purine and tryptophan metabolism were more abundant in green communities than in red communities, and were considered important in the increased growth rates of green blooms. Although we detected metabolites in these pathways, they were not identified as key determinants for either green or red blooms. This suggests that the community composition and exudates could be functionally different between Arctic and Antarctic sites. Such metabolic differences could be due to acclimation effects or true adaptation to the local environment, although more sites would need to be studied before these hypotheses can be fully tested.

### Snow algae community composition differs between bloom types but that of associated fungi and bacteria does not

The algal cells in our study were structurally similar to snow algae cells described in the Arctic (Svalbard) and North America (Hoham *et al*., 1983; Müller *et al*., 1998), with some red cells appearing morphologically similar to *Haematoccus pluvialis* (Wayama *et al*., 2013). Metabarcoding revealed that communities were dominated by an unknown *Alveolata* (SAR), a yeast in the genus *Cryptococcus* (*Tremellaceae*) and chytrids in the *Rhizophydiales* (Fungi) (Fig. 5). The *Rhizophydiales* are zoosporic fungi common in wet, cold habitats, and have been widely reported in other snow algae blooms (Schmidt *et al*., 2012; Naff *et al*., 2013; Brown *et al*., 2015; Comeau *et al*., 2016; Seto & Degawa, 2018). Within the *Chlorophyta*, we were able to detect *Chlamydomonas*,* Chlorella*, uncultured *Chloromonas* and two taxa assigned as unknown Chlorophyceae and unknown Chlorophyta. Whether the Antarctic community contains endemic species requires further study over a wider area (De Wever *et al*., 2009; Petz *et al*., 2007). In this context, Remias *et al*. (2013), studying snow algal communities from locations north (Goudier Island and Paradise Harbour, 64°S) of our location (*c. *68°S), determined species matching our metabarcoding OTUs for the red blooms (*Chloromonas*), implying that this taxon may be distributed widely, at least along the Antarctic Peninsula. In a similar study in continental Antarctica (Yatude Valley, Langhovde at 69°S), Fujii *et al*. (2010) also identified a similar community of *Chlorella*,* Chlamydomonas* and *Chloromonas* as well as other green algae (*Raphydonema* and *Koliella*) and, as here, a range of yeasts. Although the detailed composition of bacterial, protist and fungal communities in snow algal blooms may be different across the globe, it is becoming apparent that snow algae communities have similar wide functional and taxonomic structures. The dominance of fungi, specifically yeasts and chytrids, in the communities studied here is of particular note, with fungi also having been identified as important components of Arctic snow packs (Maccario *et al*., 2014). Bacteria and fungi can utilise simple and complex organic compounds within the snow pack (e.g. *Pseudomonas*, See‐Too *et al*. (2016)) and numerous studies have reported bacterial genera, such as *Polarmonas*,* Flavobacteria* and *Sphingobacteria*, living in close association with *Chloromonas* and Chlamydomonadaceae (Hoham & Duval, 2001; Komárek & Nedbalová, 2007; Hisakawa *et al*., 2015; Lutz *et al*., 2015, 2016, 2017; Hamilton & Havig, 2017). There were also a large number of OTUs that could not be assigned to a genus or species, implying that the communities in the Antarctic snow packs are yet to be fully identified, characterised and incorporated into public databases such as Silva (Quast *et al*., 2013). Additionally, the diversity of the genetic, metabolic and growth phenotypes of a wider spatial range of populations need to be determined, to assess their resilience to ongoing environmental changes and to predict future shifts in their ranges. Whether the structure of these communities will be sufficiently resilient to withstand abiotic parameters that are outside the normal range of their niche, as a result of climate change, remains to be tested by resolving the phenotypic plasticity of each species (Hoham, 1975; Morgan‐Kiss *et al*., 2006; Convey *et al*., 2014; Rengefors *et al*., 2015). Overall, such studies will contribute to understanding the functional and taxonomic diversity of polar microbial ecosystems (Keeling *et al*., 2014; Cavicchioli, 2015) and the contribution of snow algae to polar ecosystems and global carbon budgets.

## Author contributions

MPD, KKN, PC and LSP designed and planned the fieldwork and logistics. MPD carried out the fieldwork. MPD, LN and AGS planned the field sample analysis at Rothera and Cambridge with MPD and LN extracting and analysing the metabolites at Cambridge. MH‐O performed the DNA extraction for metabarcoding and PS performed the metabarcoding bioinformatics. FB and BKWL carried out the algae isolation and targeted 18S analysis. MPD and SS carried out the pigment analysis. MPD led the writing of the manuscript with all other authors contributing and editing the text. All authors have seen and approved the final version.

## Supporting information

Please note: Wiley Blackwell are not responsible for the content or functionality of any Supporting Information supplied by the authors. Any queries (other than missing material) should be directed to the *New Phytologist* Central Office.

**Fig. S1** Alpha diversity rarefaction plots.
**Fig. S2** Snow algae community biomass and cell counts.
**Fig. S3** Representative ultraviolet–visible absorption spectra of solvent ethanol extracts from green‐ or red‐dominant snow algae communities.
**Fig. S4** Pigment content of snow algae blooms.
**Fig. S5** Alpha diversity boxplots.
**Fig. S6** Screen shots of beta diversity boxplots.
**Fig. S7** Metabarcoding NMDS plots.
**Fig. S8** Composition of Chlorophyta OTUs in green and red snow algae blooms.
**Methods S1** Raw read metabarcoding R script files in vegan.
**Table S1** Sampling locations and light (PAR) and temperatures recorded at snow surface and 5 cm depth during sampling.
**Table S2** FT‐IR metabolic fingerprinting of snow algae communities.
**Table S3** Fatty acid composition of snow algae.
**Table S4** Score contribution of putatively identified metabolites associated with either green or red snow algae communities.
**Table S5** Metabolite pathways of the putatively identified metabolites associated with either green or red snow algae communities.
**Table S6** Percentage contributions and number of taxonomic assignments for Level 2 (Kingdom/Phylum) 16S rRNA gene and ITS sequences in green and red snow algae communities.
**Table S7** Percentage contributions and number of taxonomic assignments for Level 3 (Order, Class) 16S rRNA gene and ITS sequences in green and red snow algae communities.
**Table S8** Percentage contributions and numbers of taxonomic assignments for Level 6 (Family, Genus) 16S rRNA gene and ITS sequences in green and red snow algae communities.
**Table S9** Targeted genomic identification of snow algae.

## References

[nph15701-bib-0001] Altschul SF , Gish W , Miller W , Myers EW , Lipman DJ . 1990 Basic local alignment search tool. Journal of Molecular Biology 215: 403–410. 2231712 10.1016/S0022-2836(05)80360-2

[nph15701-bib-0002] Anderson MJ . 2001 A new method for non‐parametric multivariate analysis of variance. Austral Ecology 26: 32–46.

[nph15701-bib-0003] Anesio AM , Lutz S , Chrismas NAM , Benning LG . 2017 The microbiome of glaciers and ice sheets. NPJ Biofilms and Microbiomes 3: 10. 28649411 10.1038/s41522-017-0019-0 PMC5460203

[nph15701-bib-0005] Bidigare RR , Ondrusek ME . 1993 Evidence for a photoprotective function for secondary carotenoids of snow algae. Journal of Phycology 29: 427–434.

[nph15701-bib-0006] Boetius A , Anesio AM , Deming JW , Mikucki JA , Rapp JZ . 2015 Microbial ecology of the cryosphere: sea ice and glacial habitats. Nature Reviews: Microbiology 13: 677–690. 26344407 10.1038/nrmicro3522

[nph15701-bib-0007] Broady PA . 1996 Diversity, distribution and dispersal of Antarctic terrestrial algae. Biodiversity and Conservation 5: 1307–1335.

[nph15701-bib-0008] Brown SP , Olson BJSC , Jumpponen A . 2015 Fungi and algae co‐occur in snow: an issue of shared habitat or algal facilitation of heterotrophs? Arctic, Antarctic, and Alpine Research 47: 729–749.

[nph15701-bib-0009] Brunetti C , George RM , Tattini M , Field K , Davey MP . 2013 Metabolomics in plant environmental physiology. Journal of Experimental Botany 64: 4011–4020. 23922358 10.1093/jxb/ert244

[nph15701-bib-0010] Bundy JG , Davey MP , Viant MR . 2009 Environmental metabolomics: a critical review and future perspectives. Metabolomics 5: 3–21.

[nph15701-bib-0011] Burton‐Johnson A , Black M , Fretwell PT , Kaluza‐Gilbert J . 2016 An automated methodology for differentiating rock from snow, clouds and sea in Antarctica from Landsat 8 imagery: a new rock outcrop map and area estimation for the entire Antarctic continent. Cryosphere 10: 1665–1677.

[nph15701-bib-0012] Callahan BJ , McMurdie PJ , Rosen MJ , Han AW , Johnson AJA , Holmes SP . 2016 DADA2: high‐resolution sample inference from Illumina amplicon data. Nature Methods 13: 581–583. 27214047 10.1038/nmeth.3869 PMC4927377

[nph15701-bib-0013] Caporaso JG , Kuczynski J , Stombaugh J , Bittinger K , Bushman FD , Costello EK , Fierer N , Pena AG , Goodrich JK , Gordon JI *et al* 2010 QIIME allows analysis of high‐throughput community sequencing data. Nature Methods 7: 335–336. 20383131 10.1038/nmeth.f.303 PMC3156573

[nph15701-bib-0014] Cavicchioli R . 2015 Microbial ecology of Antarctic aquatic systems. Nature Reviews: Microbiology 13: 691–706. 26456925 10.1038/nrmicro3549

[nph15701-bib-0015] Chong J , Soufan O , Li C , Caraus I , Li S , Bourque G , Wishart DS , Xia J . 2018 MetaboAnalyst 4.0: towards more transparent and integrative metabolomics analysis. Nucleic Acids Research 46: W486–W494. 29762782 10.1093/nar/gky310 PMC6030889

[nph15701-bib-0016] Chong J , Xia J . 2018 MetaboAnalystR: an R package for flexible and reproducible analysis of metabolomics data. Bioinformatics 34: 4313–4314. 29955821 10.1093/bioinformatics/bty528 PMC6289126

[nph15701-bib-0017] Chown SL , Convey P . 2012 Spatial and temporal variability in terrestrial Antarctic biodiversity In: Rogers AD, Johnston NM, Murphy EJ, Clarke A, eds. Antarctic ecosystems: an extreme environment in a changing world. Chichester, UK: Blackwell Publishing Ltd, 13–43.

[nph15701-bib-0018] Comeau AM , Vincent WF , Bernier L , Lovejoy C . 2016 Novel chytrid lineages dominate fungal sequences in diverse marine and freshwater habitats. Scientific Reports 6: 30120. 27444055 10.1038/srep30120 PMC4957111

[nph15701-bib-0019] Convey P . 2011 Antarctic terrestrial biodiversity in a changing world. Polar Biology 34: 1629–1641.

[nph15701-bib-0020] Convey P . 2017 Antarctic biodiversity. Reference module in life sciences. Elsevier. [WWW document] URL 10.1016/B978-0-12-809633-8.02182-8 [accessed 9 January 2019].

[nph15701-bib-0021] Convey P , Chown SL , Clarke A , Barnes DKA , Bokhorst S , Cummings V , Ducklow HW , Frati F , Green TGA , Gordon S *et al* 2014 The spatial structure of Antarctic biodiversity. Ecological Monographs 84: 203–244.

[nph15701-bib-0022] Cook JM , Hodson AJ , Taggart AJ , Mernild SH , Tranter M . 2017 A predictive model for the spectral “bioalbedo” of snow. Journal of Geophysical Research: Earth Surface 122: 434–454.

[nph15701-bib-0023] Davey MP , Burrell MM , Woodward FI , Quick WP . 2008 Population specific metabolic phenotypes of *Arabidopsis lyrata* ssp. *petraea* . New Phytologist 177: 380–388. 18028292 10.1111/j.1469-8137.2007.02282.x

[nph15701-bib-0024] Davey MP , Duong GH , Tomsett E , Litvinenko ACP , Howe CJ , Horst I , Smith AG . 2014 Triacylglyceride production and autophagous responses in *Chlamydomonas reinhardtii* depend on resource allocation and carbon source. Eukaryotic Cell 13: 392–400. 24413660 10.1128/EC.00178-13 PMC3957581

[nph15701-bib-0025] De Wever A , Leliaert F , Verleyen E , Vanormelingen P , Van der Gucht K , Hodgson DA , Sabbe K , Vyverman W . 2009 Hidden levels of phylodiversity in Antarctic green algae: further evidence for the existence of glacial refugia. Proceedings of the Royal Society of London B: Biological Sciences 276: 3591–3599. 10.1098/rspb.2009.0994 PMC2817313 19625320

[nph15701-bib-0026] Demmig‐Adams B , Adams WW . 1996 The role of xanthophyll cycle carotenoids in the protection of photosynthesis. Trends in Plant Science 1: 21–26.

[nph15701-bib-0027] Dierssen HM , Smith RC , Vernet M . 2002 Glacial meltwater dynamics in coastal waters west of the Antarctic Peninsula. Proceedings of the National Academy of Sciences, USA 99: 1790–1795. 10.1073/pnas.032206999 PMC122272 11830636

[nph15701-bib-0028] Dunn WB , Broadhurst D , Begley P , Zelena E , Francis‐McIntyre S , Anderson N , Brown M , Knowles JD , Halsall A , Haselden JN *et al* 2011 Procedures for large‐scale metabolic profiling of serum and plasma using gas chromatography and liquid chromatography coupled to mass spectrometry. Nature Protocols 6: 1060–1083. 21720319 10.1038/nprot.2011.335

[nph15701-bib-0029] Eggert A , Karsten U . 2010 Low molecular weight carbohydrates in red algae – an ecophysiological and biochemical perspective In: Seckbach J, Chapman D, eds. Cellular origin, life in extreme habitats and astrobiology: red algae in the genomics age. Dordrecht, the Netherlands: Springer Science and Business Media, V13 443–456.

[nph15701-bib-0030] Fazius F , Shelest E , Gebhardt P , Brock M . 2012 The fungal α‐aminoadipate pathway for lysine biosynthesis requires two enzymes of the aconitase family for the isomerization of homocitrate to homoisocitrate. Molecular Microbiology 86: 1508–1530. 23106124 10.1111/mmi.12076 PMC3556520

[nph15701-bib-0031] Fogg GE . 1967 Observations on the snow algae of the South Orkney Islands. Philosophical Transactions of the Royal Society of London. Series B: Biological Sciences 252: 279–287.

[nph15701-bib-0032] Fretwell PT , Convey P , Fleming AH , Peat HJ , Hughes KA . 2011 Detecting and mapping vegetation distribution on the Antarctic Peninsula from remote sensing data. Polar Biology 34: 273–281.

[nph15701-bib-0033] Fujii M , Takano Y , Kojima H , Hoshino T , Tanaka R , Fukui M . 2010 Microbial community structure, pigment composition, and nitrogen source of red snow in Antarctica. Microbial Ecology 59: 466–475. 19847476 10.1007/s00248-009-9594-9 PMC4261141

[nph15701-bib-0034] Ganey GQ , Loso MG , Bryant Burgess A , Dial RJ . 2017 The role of microbes in snowmelt and radiative forcing on an Alaskan icefield. Nature Geoscience 10: 754–759.

[nph15701-bib-0035] Goldmann A , Message B , Tepfer D , Molyneux RJ , Duclos O , Boyer FD , Pan YT , Elbein AD . 1996 Biological activities of the nortropane alkaloid, calystegine B_2_, and analogs: structure–function relationships. Journal of Natural Products 59: 1137–1142. 8988598 10.1021/np960409v

[nph15701-bib-0036] Hamilton TL , Havig J . 2017 Primary productivity of snow algae communities on stratovolcanoes of the Pacific Northwest. Geobiology 15: 280–295. 27917584 10.1111/gbi.12219 PMC5324535

[nph15701-bib-0037] Hisakawa N , Quistad SD , Hester ER , Martynova D , Maughan H , Sala E , Gavrilo MV , Rohwer F . 2015 Metagenomic and satellite analyses of red snow in the Russian Arctic. PeerJ 3: e1491. 26713242 10.7717/peerj.1491 PMC4690372

[nph15701-bib-0038] Hodson A , Anesio AM , Tranter M , Fountain A , Osborn M , Priscu J , Laybourn‐Parry J , Sattler B . 2008 Glacial ecosystems. Ecological Monographs 78: 41–67.

[nph15701-bib-0039] Hoham RW . 1975 Optimum temperatures and temperature ranges for growth of snow algae. Arctic and Alpine Research 7: 13–24.

[nph15701-bib-0040] Hoham RW , Duval B . 2001 Microbial ecology of snow and freshwater ice with emphasis on snow algae In: Jones HG, Pomeroy JW, Walker DA, Hoham RW, eds. Snow ecology: an interdisciplinary examination of snow‐covered ecosystems. Cambridge, UK: Cambridge University Press, 168–228.

[nph15701-bib-0041] Hoham RW , Mullet JE , Roemer SC . 1983 The life history and ecology of the snow alga *Chloromonas polyptera* comb. nov. (Chlorophyta, Volvocales). Canadian Journal of Botany 61: 2416–2429.

[nph15701-bib-0042] Holzinger A , Allen MC , Dimitri DD . 2016 Hyperspectral imaging of snow algae and green algae from aeroterrestrial habitats. Journal of Photochemistry & Photobiology, B: Biology 162: 412–420. 10.1016/j.jphotobiol.2016.07.001 PMC5061078 27442511

[nph15701-bib-0043] Inskeep WP , Bloom PR . 1985 Extinction coefficients of chlorophyll‐a and chlorophyll‐b in n, n‐dimethylformamide and 80‐percent acetone. Plant Physiology 77: 483–485. 16664080 10.1104/pp.77.2.483 PMC1064541

[nph15701-bib-0044] Keeling PJ , Burki F , Wilcox HM , Allam B , Allen EE , Amaral‐Zettler LA , Armbrust EV , Archibald JM , Bharti AK , Bell CJ *et al* 2014 The Marine Microbial Eukaryote Transcriptome Sequencing Project (MMETSP): illuminating the functional diversity of eukaryotic life in the oceans through transcriptome sequencing. PLoS Biology 12: e1001889. 24959919 10.1371/journal.pbio.1001889 PMC4068987

[nph15701-bib-0045] Komárek J , Nedbalová L . 2007 Green cryosestic algae In: Seckbach J, ed. Algae and cyanobacteria in extreme environments. Dordrecht, the Netherlands: Springer, 321–342.

[nph15701-bib-0046] Leya T . 2013 Snow algae: adaptation strategies to survive on snow and ice In: Seckbach J, Oren A, Stan‐Lotter H, eds. Polyextremophiles. Cellular origin, life in extreme habitats and astrobiology, vol. 27. Dordrecht, the Netherlands: Springer, 401–423.

[nph15701-bib-0047] Lukeš M , Procházková L , Shmidt V , Nedbalová L , Kaftan D . 2014 Temperature dependence of photosynthesis and thylakoid lipid composition in the red snow alga *Chlamydomonas* cf. *nivalis* (Chlorophyceae). FEMS Microbiology Ecology 89: 303–315. 24698015 10.1111/1574-6941.12299

[nph15701-bib-0048] Lutz S , Anesio AM , Edwards R , Benning LG . 2017 Linking microbial diversity and functionality of arctic glacial surface habitats. Environmental Microbiology 19: 551–565. 27511455 10.1111/1462-2920.13494

[nph15701-bib-0049] Lutz S , Anesio AM , Field K , Benning LG . 2015 Integrated ‘Omics’, targeted metabolite and single‐cell analyses of Arctic snow algae functionality and adaptability. Frontiers in Microbiology 6: 1323. 26635781 10.3389/fmicb.2015.01323 PMC4659291

[nph15701-bib-0050] Lutz S , Anesio AM , Jorge Villar SE , Benning LG . 2014 Variations of algal communities cause darkening of a Greenland glacier. FEMS Microbiology Ecology 89: 402–414. 24920320 10.1111/1574-6941.12351

[nph15701-bib-0051] Lutz S , Anesio AM , Raiswell R , Edwards A , Newton RJ , Gill F , Benning LG . 2016 The biogeography of red snow microbiomes and their role in melting arctic glaciers. Nature Communications 7: 11968. 10.1038/ncomms11968 PMC4917964 27329445

[nph15701-bib-0052] Maccario L , Vogel TM , Larose C . 2014 Potential drivers of microbial community structure and function in Arctic spring snow. Frontiers in Microbiology 5: Article 413. 25147550 10.3389/fmicb.2014.00413 PMC4124603

[nph15701-bib-0054] Minhas AK , Hodgson P , Barrow CJ , Adholeya A . 2016 A review on the assessment of stress conditions for simultaneous production of microalgal lipids and carotenoids. Frontiers in Microbiology 7: 546. 27199903 10.3389/fmicb.2016.00546 PMC4853371

[nph15701-bib-0055] Morgan‐Kiss RM , Priscu JC , Pocock T , Gudynaite‐Savitch L , Huner NPA . 2006 Adaptation and acclimation of photosynthetic microorganisms to permanently cold environments. Microbiology and Molecular Biology Reviews 70: 222–252. 16524924 10.1128/MMBR.70.1.222-252.2006 PMC1393254

[nph15701-bib-0056] Müller T , Bleiβ W , Martin CD , Rogaschewski S , Fuhr G . 1998 Snow algae from northwest Svalbard: their identification, distribution, pigment and nutrient content. Polar Biology 20: 14–32.

[nph15701-bib-0057] Naff CS , Darcy JL , Schmidt SK . 2013 Phylogeny and biogeography of an uncultured clade of snow chytrids. Environmental Microbiology 15: 2672–2680. 23551529 10.1111/1462-2920.12116

[nph15701-bib-0058] Paliy O , Shankar V . 2016 Application of multivariate statistical techniques in microbial ecology. Molecular Ecology 25: 1032–1057. 26786791 10.1111/mec.13536 PMC4769650

[nph15701-bib-0059] Petz W , Valbonesi A , Schiftner U , Quesada A , Cynan Ellis‐Evans J . 2007 Ciliate biogeography in Antarctic and Arctic freshwater ecosystems: endemism or global distribution of species? FEMS Microbiology Ecology 59: 396–408. 17313584 10.1111/j.1574-6941.2006.00259.x

[nph15701-bib-0060] Quast C , Pruesse E , Yilmaz P , Gerken J , Schweer T , Yarza P , Peplies J , Glöckner FO . 2013 The SILVA ribosomal RNA gene database project: improved data processing and web‐based tools. Nucleic Acids Research 41: D590–D596. 23193283 10.1093/nar/gks1219 PMC3531112

[nph15701-bib-0061] Remias D , Albert A , Lütz C . 2010 Effects of realistically simulated, elevated UV irradiation on photosynthesis and pigment composition of the alpine snow alga *Chlamydomonas nivalis* and the Arctic soil alga *Tetracystis* sp. (Chlorophyceae). Photosynthetica 48: 269–277.

[nph15701-bib-0062] Remias D , Lütz C . 2007 Characterisation of esterified secondary carotenoids and of their isomers in green algae: a HPLC approach. Algological Studies 124: 85–94.

[nph15701-bib-0063] Remias D , Lütz‐Meindl U , Lütz C . 2005 Photosynthesis, pigments and ultrastructure of the alpine snow alga *Chlamydomonas nivalis* . European Journal of Phycology 40: 259–268.

[nph15701-bib-0064] Remias D , Wastian H , Lütz C , Leya T . 2013 Insights into the biology and phylogeny of *Chloromonas polyptera* (Chlorophyta), an alga causing orange snow in Maritime Antarctica. Antarctic Science 25: 648–656.

[nph15701-bib-0065] Rengefors K , Logares R , Laybourn‐Parry J , Gast RJ . 2015 Evidence of concurrent local adaptation and high phenotypic plasticity in a polar microeukaryote. Environmental Microbiology 17: 1510–1519. 25041758 10.1111/1462-2920.12571

[nph15701-bib-0066] Řezanka T , Nedbalová L , Procházková L , Sigler K . 2014 Lipidomic profiling of snow algae by ESI‐MS and silver‐LC/APCI‐MS. Phytochemistry 100: 34–42. 24548555 10.1016/j.phytochem.2014.01.017

[nph15701-bib-0067] Rintoul SR , Chown SL , De Conto RM , England MH , Fricker HA , Masson‐Delmotte V , Naish TR , Siegert MJ , Xavier JC . 2018 Choosing the future of Antarctica. Nature 558: 233–241. 29899481 10.1038/s41586-018-0173-4

[nph15701-bib-0068] Rogers AD , Clarke A , Johnston NM , Murphy EJ . 2007 Introduction. Antarctic ecology from genes to ecosystems: the impact of climate change and the importance of scale. Philosophical Transactions of the Royal Society of London. Series B: Biological Sciences 362: 5–9. 17405205 10.1098/rstb.2006.1943 PMC1764835

[nph15701-bib-0069] Schmidt SK , Naff CS , Lynch RC . 2012 Fungal communities at the edge: ecological lessons from high alpine fungi. Fungal Ecology 5: 443–452.

[nph15701-bib-0070] See‐Too WS , Lima Ee R , Convey P , Pearce DA , Yin WF , Chan KG . 2016 Complete genome of *Pseudomonas* sp. strain L10.10, a psychrotolerant biofertilizer that could promote plant growth. Journal of Biotechnology 222: 84–85. 26876481 10.1016/j.jbiotec.2016.02.017

[nph15701-bib-0071] Seto K , Degawa Y . 2018 *Collimyces mutans* gen. et sp. nov. (Rhizophydiales, Collimycetaceae fam. nov.), a new chytrid parasite of Microglena (Volvocales, clade Monadinia). Protist 169: 507–520. 29935342 10.1016/j.protis.2018.02.006

[nph15701-bib-0072] Spijkerman E , Wacker A , Weithoff G , Leya T . 2012 Elemental and fatty acid composition of snow algae in Arctic habitats. Frontiers in Microbiology 3: 390. 23112797 10.3389/fmicb.2012.00380 PMC3482990

[nph15701-bib-0073] Stibal M , Box JE , Cameron KA , Langen PL , Yallop ML , Mottram RH , Khan AL , Molotch NP , Chrismas NAM , Calì Quaglia F *et al* 2017 Algae drive enhanced darkening of bare ice on the Greenland ice sheet. Geophysical Research Letters 44: 11463–11471.

[nph15701-bib-0074] Turner J , Bindschadler R , Convey P , di Prisco G , Fahrbach E , Gutt J , Hodgson D , Mayewski P , Summerhayes C . 2009 Antarctic climate change and the environment. Cambridge, UK: Scientific Committee on Antarctic Research.

[nph15701-bib-0075] Turner J , Lu H , White I , King JC , Phillips T , Hosking JS , Bracegirdle TJ , Marshall GJ , Mulvaney R , Deb P . 2016 Absence of 21^st^ century warming on Antarctic Peninsula consistent with natural variability. Nature 535: 411–415. 27443743 10.1038/nature18645

[nph15701-bib-0076] Vaughan DG . 2006 Recent trends in melting conditions on the Antarctic Peninsula and their implications of ice‐sheet mass balance and sea level. Arctic, Antarctic, and Alpine Research 38: 147–152.

[nph15701-bib-0077] Vyverman W , Verleyen E , Wilmotte A , Hodgson DA , Willems A , Peeters K , Van de Vijver B , De Wever A , Leliaert F , Sabbe K . 2010 Evidence for widespread endemism among Antarctic micro‐organisms. Polar Science 4: 103–113.

[nph15701-bib-0078] Wang Y , Tian RM , Gao ZM , Bougouffa S , Qian PY . 2014 Optimal eukaryotic 18S and universal 16S/18S ribosomal RNA primers and their application in a study of symbiosis. PLoS ONE 9: e90053. 24594623 10.1371/journal.pone.0090053 PMC3940700

[nph15701-bib-0079] Wayama M , Ota S , Matsuura H , Nango N , Hirata A , Kawano S . 2013 Three‐dimensional ultrastructural study of oil and astaxanthin accumulation during encystment in the green alga *Haematococcus pluvialis* . PLoS ONE 8: e53618. 23326471 10.1371/journal.pone.0053618 PMC3543331

[nph15701-bib-0080] Wellburn AR . 1994 The spectral determination of chlorophyll‐*a* and chlorophhyll‐*b*, as well as total carotenoids, using various solvents with spectrophotometers of different resolution. Journal of Plant Physiology 144: 307–313.

[nph15701-bib-0081] Williams WE , Gorton HL , Vogelmann TC . 2003 Surface gas‐exchange processes of snow algae. Proceedings of the National Academy of Sciences, USA 100: 562–566. 10.1073/pnas.0235560100 PMC141035 12518048

