# Supplementary material for: Snow algae communities in Antarctica: metabolic and taxonomic composition
Source: New Phytol. 2019 Feb 27;222(3):1242–55. doi: 10.1111/nph.15701 (PMC6492300; doi:10.1111/nph.15701)
Supplement: Supplementary file 1 — Fig. S1 Alpha diversity rarefaction plots. Fig. S2 Snow algae community biomass and cell counts. Fig. S3 Representative ultraviolet–visible absorption spectra of solvent ethanol extracts from green‐ or red‐dominant snow algae communities. Fig. S4 Pigment content of snow algae blooms. Fig. S5 Alpha diversity boxplots. Fig. S6 Screen shots of beta diversity boxplots. Fig. S7 Metabarcoding NMDS plots. Fig. S8 Composition of Chlorophyta OTUs in green and red snow algae blooms. Methods S1 Raw read metabarcoding R script files in vegan. Table S1 Sampling locations and light (PAR) and temperatures recorded at snow surface and 5 cm depth during sampling. Table S2 FT‐IR metabolic fingerprinting of snow algae communities. Table S3 Fatty acid composition of snow algae. Table S4 Score contribution of putatively identified metabolites associated with either green or red snow algae communities. Table S5 Metabolite pathways of the putatively identified metabolites associated with either green or red snow algae communities. Table S6 Percentage contributions and number of taxonomic assignments for Level 2 (Kingdom/Phylum) 16S rRNA gene and ITS sequences in green and red snow algae communities. Table S7 Percentage contributions and number of taxonomic assignments for Level 3 (Order, Class) 16S rRNA gene and ITS sequences in green and red snow algae communities. Table S8 Percentage contributions and numbers of taxonomic assignments for Level 6 (Family, Genus) 16S rRNA gene and ITS sequences in green and red snow algae communities. Table S9 Targeted genomic identification of snow algae. [file NPH-222-1242-s001.pdf]

New Phytologist **Supporting Information**

Article title: **Snow algae communities in Antarctica - metabolic and taxonomic composition**

Authors: Matthew P. Davey, Louisa Norman, Peter Sterk, Maria Huete-Ortega, Freddy Bunbury, Bradford Kin Wai Loh, Sian Stockton, Lloyd S. Peck, Peter Convey, Kevin K. Newsham, Alison G. Smith

Article acceptance date: 08 January 2019

The following Supporting Information is available for this article:

Fig. S1 Supporting Information Figure S1: Alpha diversity rarefaction plots (to determine if the richness of the samples has been fully observed or sequenced) was calculated using the QIIME [diversity alpha-rarefaction] visualiser platform (Supporting QIIME script 3) for (a) 16S and (b) 18S sequence data from green and red communities.

(a) 16S

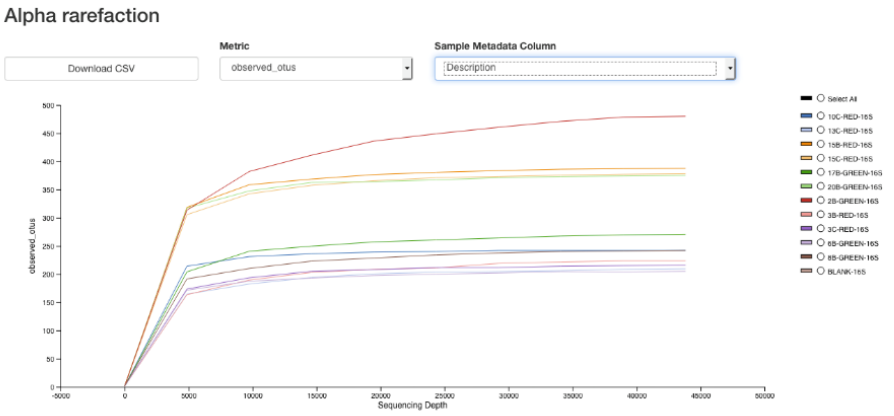

(b) ITS (18S)

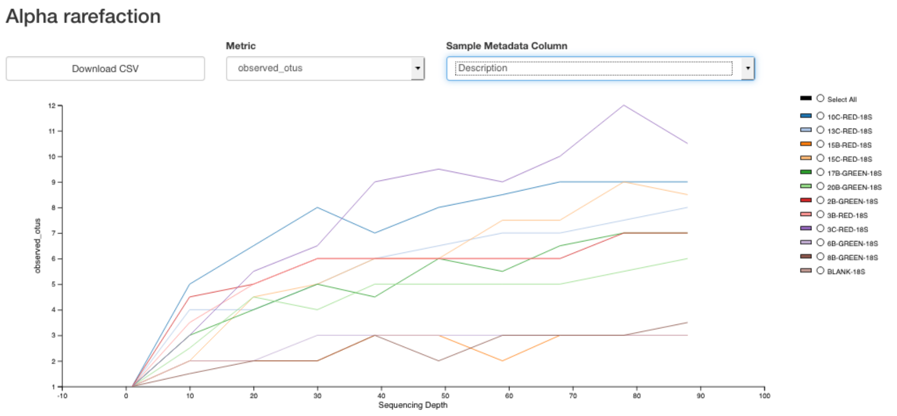

Fig. S2 Supporting Information Figure S2: Snow algae community biomass as (a) cell number (cells ml-1 snow melt) and (b) dry cell mass (mg l-1 snow melt) from green and red snow algae communities collected from four locations adjacent to the Antarctic Peninsula (Rothera Point (RP), Anchorage Island (AN), Léonie Island (LE) and Lagoon Island (LG)) during January and February 2015 (austral summer). Data are mean ± SD, Green, red sample sizes (n) for cell numbers are: RP 25,16; AN 15,30; LE 4,4; LG 12,14 and for dry weight are: RP 13,9; AN 9,18; LE 3,6; LG 9,8. Statistical differences (ANOVA) between green and red communities within a location are denoted by * = P ⩽ 0.05, *** = P ⩽ 0.001.

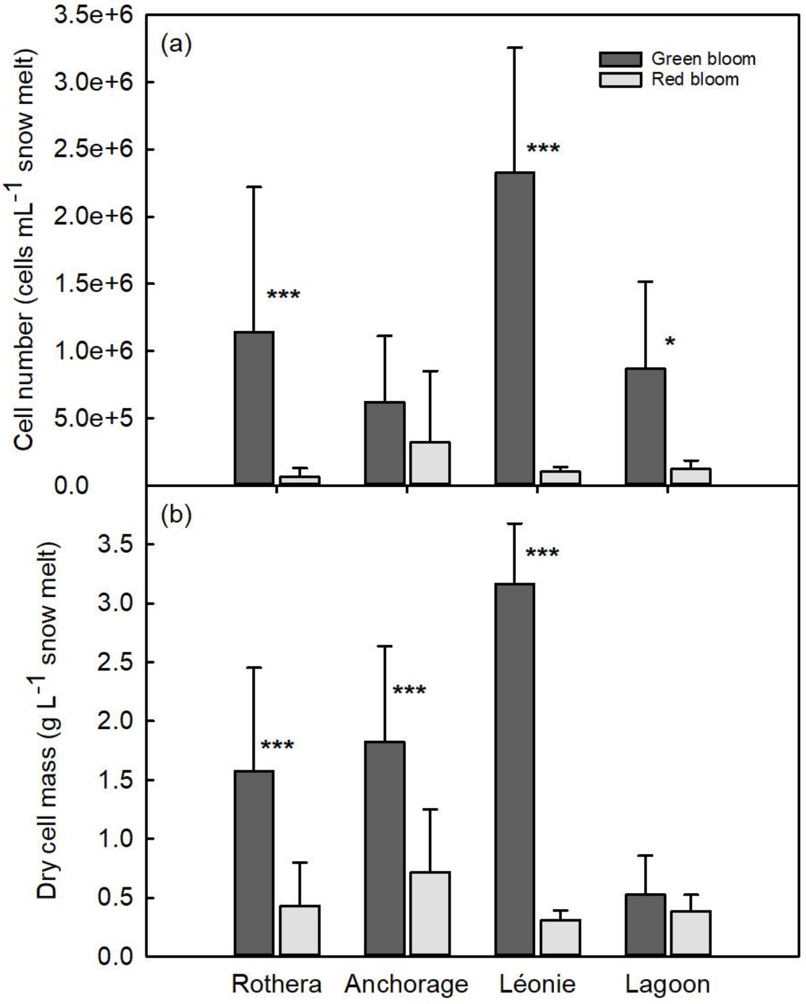

Fig. S3 Supporting Information Figure S3: Representative UV-Vis absorption spectra of solvent ethanol extracts from green or red dominant snow algae communities taken within 24 h of collection from the field. Peaks associated with chlorophyll a (Chl a), chlorophyll b (Chl b) and astaxanthin (λmax = 475 nm) are highlighted. Representative green and red phase snow algal cells are also shown (~10 µm cell diameter).

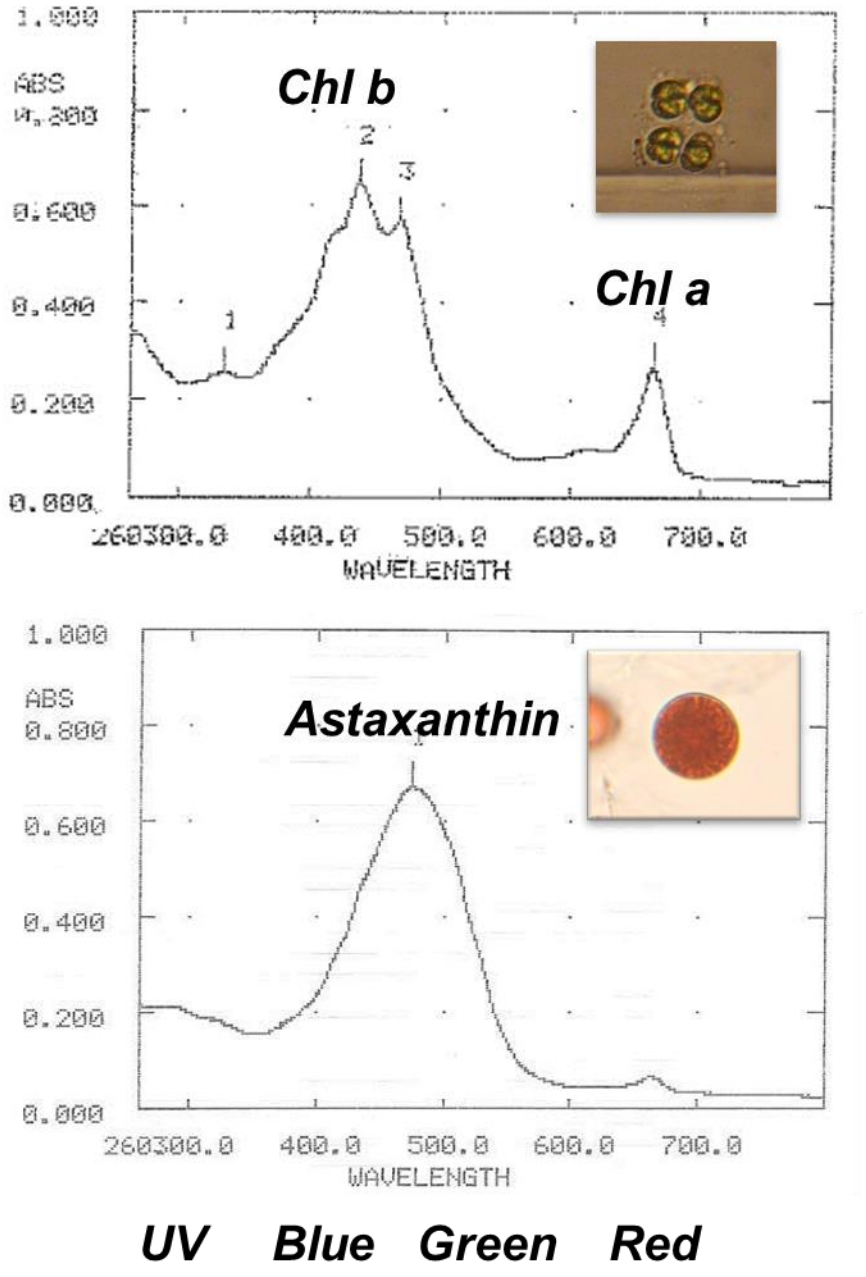

Fig. S4 Supporting Information Figure S4: Pigment content of snow algae blooms. Total chlorophyll and carotenoids expressed as both mg g-1 dry cell mass (DCM; panels A and C) and mg l-1 snow melt (panels B and D) from green and red snow algae communities collected from four locations adjacent to the Antarctic Peninsula (Rothera Point (RP), Anchorage Island (AN), Léonie Island (LE) and Lagoon Island (LG)) during January and February 2015 (austral summer). Data are mean ± SD. Data are mean ± SD. Green, red sample sizes (n) = RP 4,3; AN 3,6; LE 1,2; LG 3,3. Statistical differences (ANOVA) between green and red communities within a location are denoted by * = P ⩽ 0.05, ** = P ⩽ 0.01, *** = P ⩽ 0.001. None of the carotenoid measurements were statistically different between bloom colours.

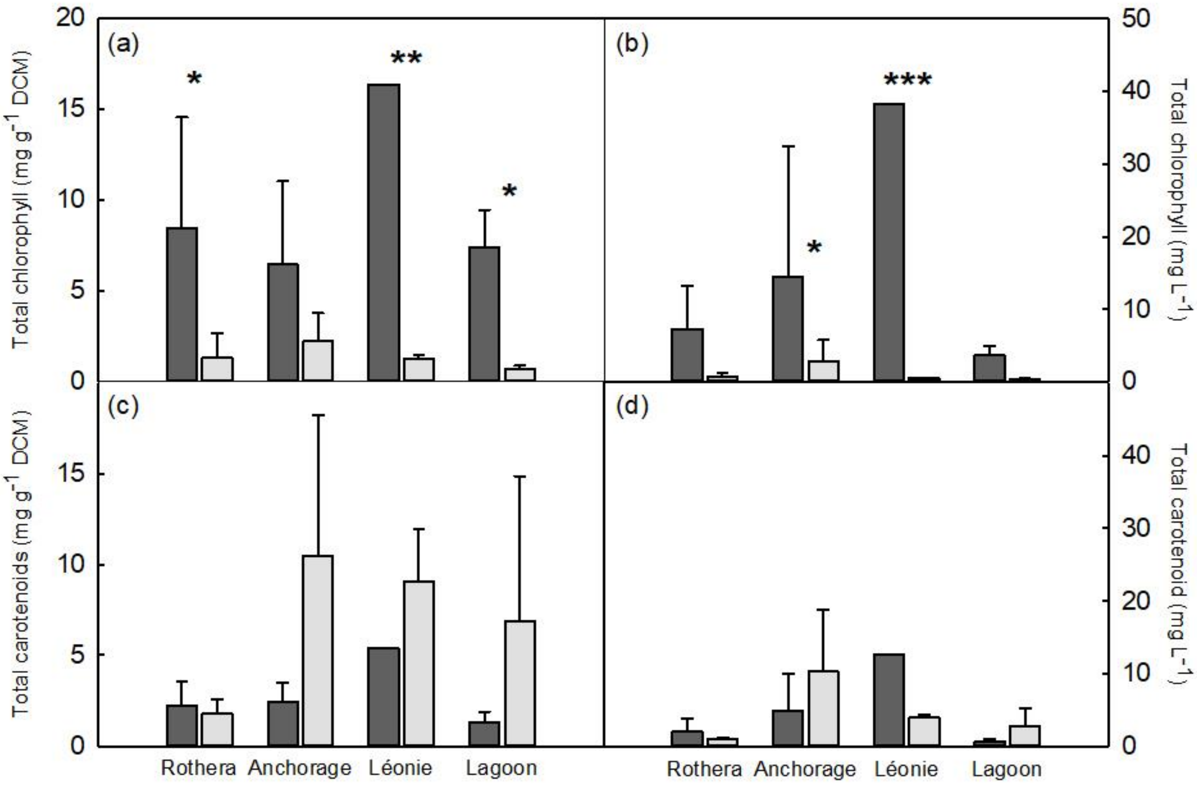

Fig. S5 Supporting Information Figure S5: Screen shots of the alpha diversity boxplots to test for associations between discrete metadata categories (green and red algae communities) and 16S (a, b) and ITS (18S) (c, d) alpha diversity data using Faith's Phylogenetic Diversity (a qualitative measure of community richness that incorporates phylogenetic relationships between the features) and Pielou's Evenness (a measure of community evenness). Plots and H and P values Kruskall-Wallis test) were measured using QIIME 2's diversity analyses [q2-diversity] plugin essentially as described in QIIME 2's moving pictures tutorial (version 2017.10; https://docs.qiime2.org/2017.10/tutorials/moving-pictures/).

	Faith's Phylogenetic Diversity	Pielou's Evenness
16S	(a) (H = 0.13, *P* = 0.72) 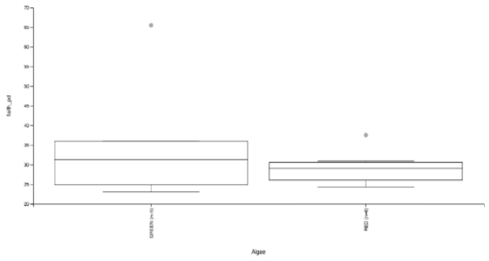	(b) (H = 0.03, *P* = 0.86) 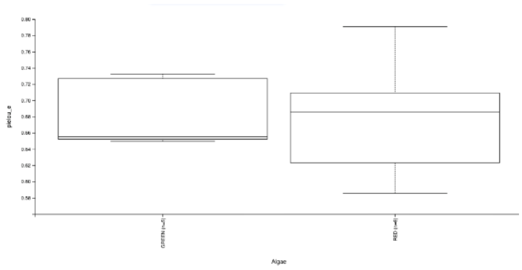
ITS	(c) (H = 1.20, *P* = 0.27) 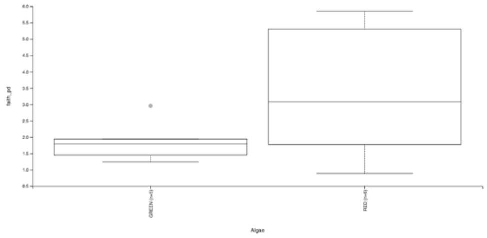	(d) (H = 0.03, *P* = 0.86) 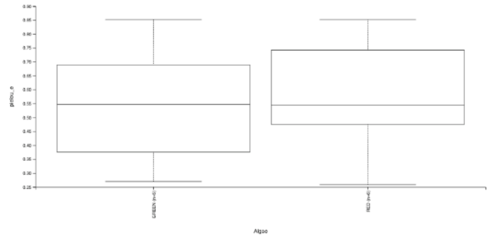

Fig. S6 Supporting Information Figure S6: Screen shots of beta diversity boxplots using PERMANOVA to test for within and between green and red bloom community dissimilarity on 16S (a, b) and ITS (18S) (c, d) sequences. Plots and P values ($P \geqslant 0.05$, Kruskall-Wallis test) were measured using QIIME 2's diversity analyses [q2-diversity] plugin essentially as described in QIIME 2's moving pictures tutorial (version 2017.10; ttps://docs.qiime2.org/2017.10/tutorials/moving-pictures/).

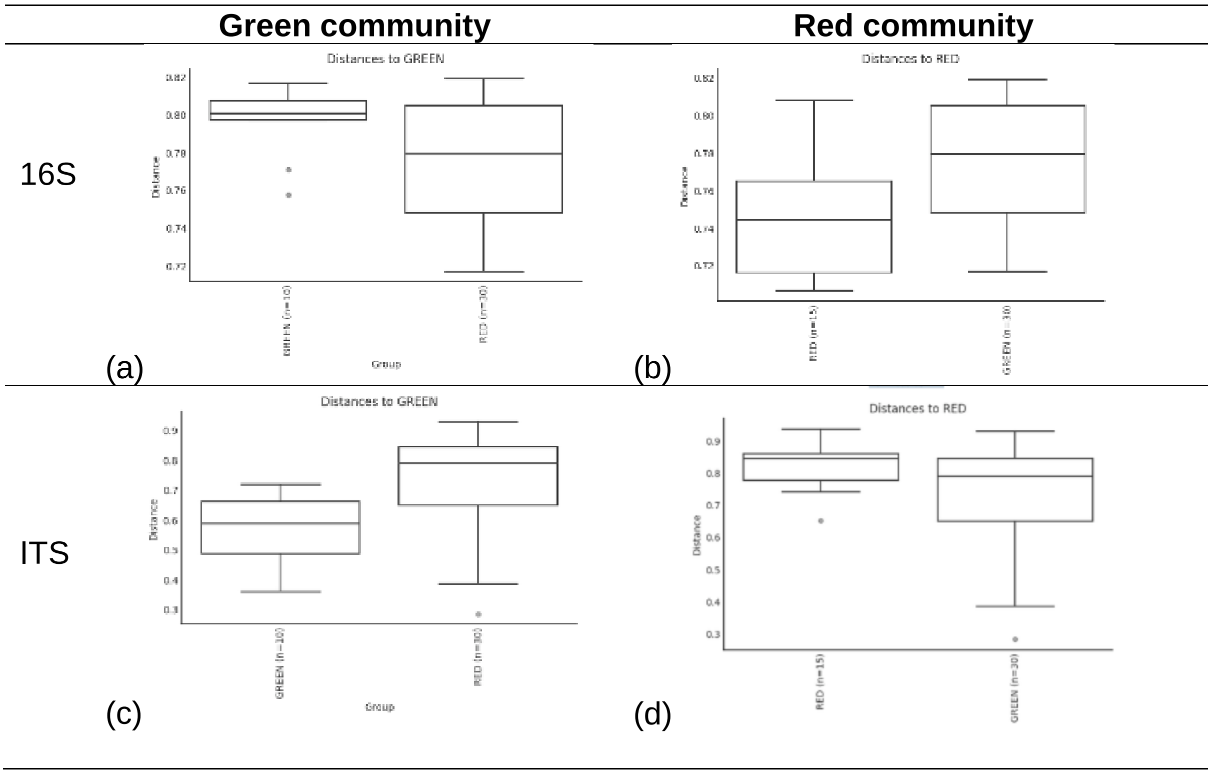

Fig. S7 Supporting Information Figure S7: Metabarcoding reveals taxonomic similarities between green and red blooms. NMDS plots from raw taxonomically assigned 16S and ITS (18S) OTU sequence reads, and all OTUs detected (ignoring taxonomic assignments and 16S and ITS (18S) OTUs combined) of green and red snow algae communities collected from four locations adjacent to the Antarctic Peninsula (Rothera Point (RP), Anchorage Island (AN), Léonie Island (LE) and Lagoon Island (LA)) during January and February 2015 (austral summer). Polygons were added based on community colour for clarification. Plots were calculated using R script in vegan (Supporting data R script; https://jonlefcheck.net/2012/10/24/nmds-tutorial-in-r/) with stress levels 0.1-0.2, using the Bray-Curtis dissimilarity calculation (Paliy & Shankar 2016).

	16S	18S
**CLASS**	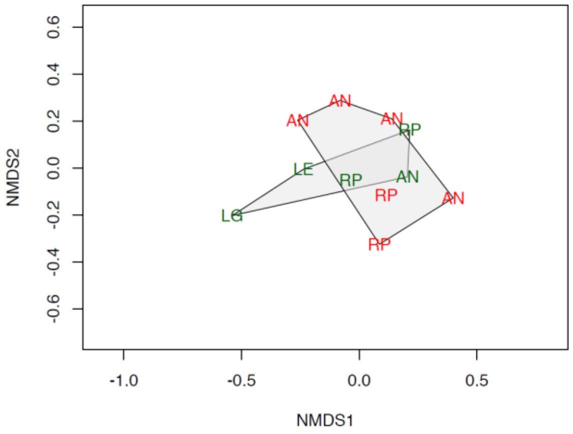	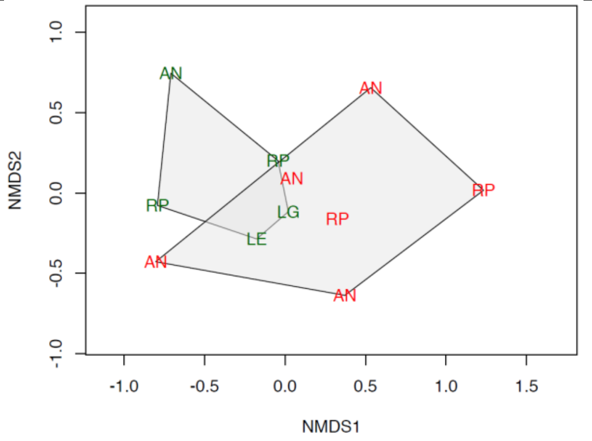
**GENUS**	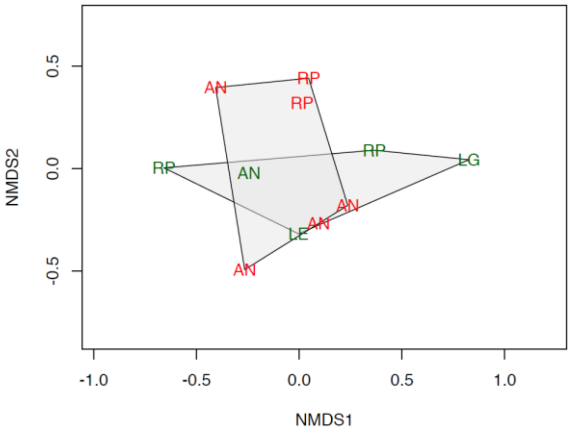	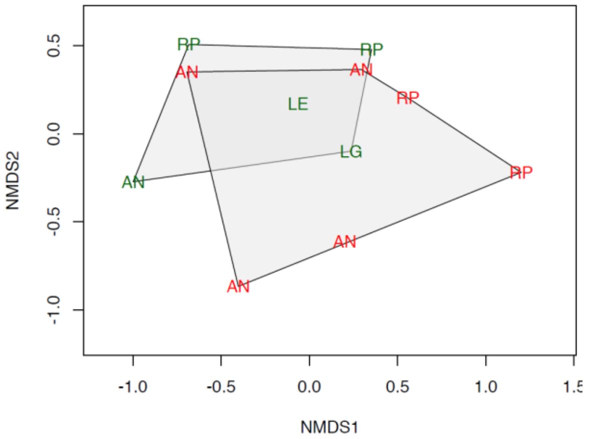
**All OTUs**	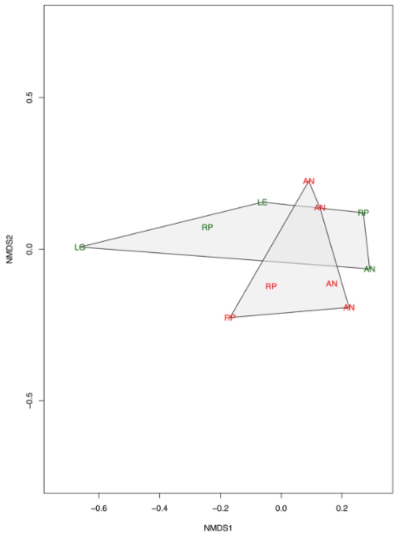	

Fig. S8 Supporting Information Figure S8: Composition of Chlorophyta OTUs in green and red snow algae blooms. Percent contribution of taxonomic assignments for 99% aligned OTUs for ITS1 (18S) sequences in green and red snow algae communities from Rothera Point, Anchorage Island, Léonie Island and Lagoon Island in Ryder Bay, Antarctica during January and February 2015 (austral summer). Percent contribution values are the mean relative abundance of the taxa in percentage of total sequences with more than 0.5% abundance and are classified at the Chlorophyta level or below. All values are mean of n = 5 (green community sites) and n = 6 (red community sites).

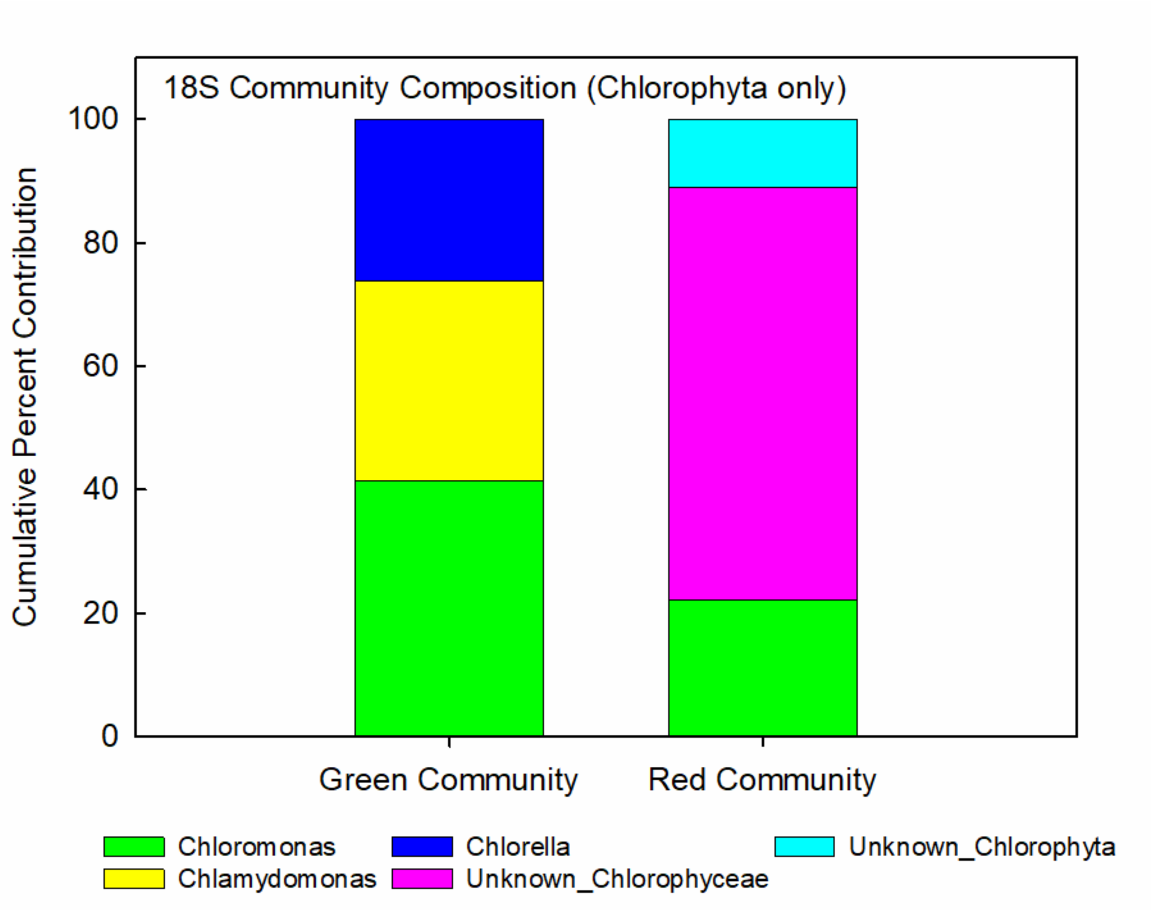

Table S1 Supporting Information Table S1: Sampling island locations, average (± SD) light (PAR) received at snow surfaces and temperatures (snow surface and 5 cm depth) recorded during sampling (Jan–Feb 2015) in Ryder Bay, Antarctic Peninsula. Green and red blooms were sampled from all locations. Blooms lasted for at least 42 d, with bloom areas ranging from approximately 5 m2 to >2500 m2. n.d. = no data.

Location	Latitude/Longitude	Number of blooms studied	PAR µmol m^{-2} s^{-1}	Temperature °C (surface)	Temperature °C (5cm depth)
Rothera Point	67°34'05.9"S 68°06'50.7"W	7	684 ± 224	1.4 ± 1.6	0.06 ± 0.03
Anchorage Island	67°36'15.9"S 68°12'36.9"W	9	1118 ± 576	0.1 ± 0.06	0.06 ± 0.03
Lagoon Island	67°35'36.7"S 68°14'18.6"W	10	n.d.	0.08 ± 3.88	-3.83 ± 0.41
Léonie Island	67°35'52.3"S 68°19'32.0"W	5	1271 ± 256	-0.17 ± 3.18	-0.67 ± 3.21

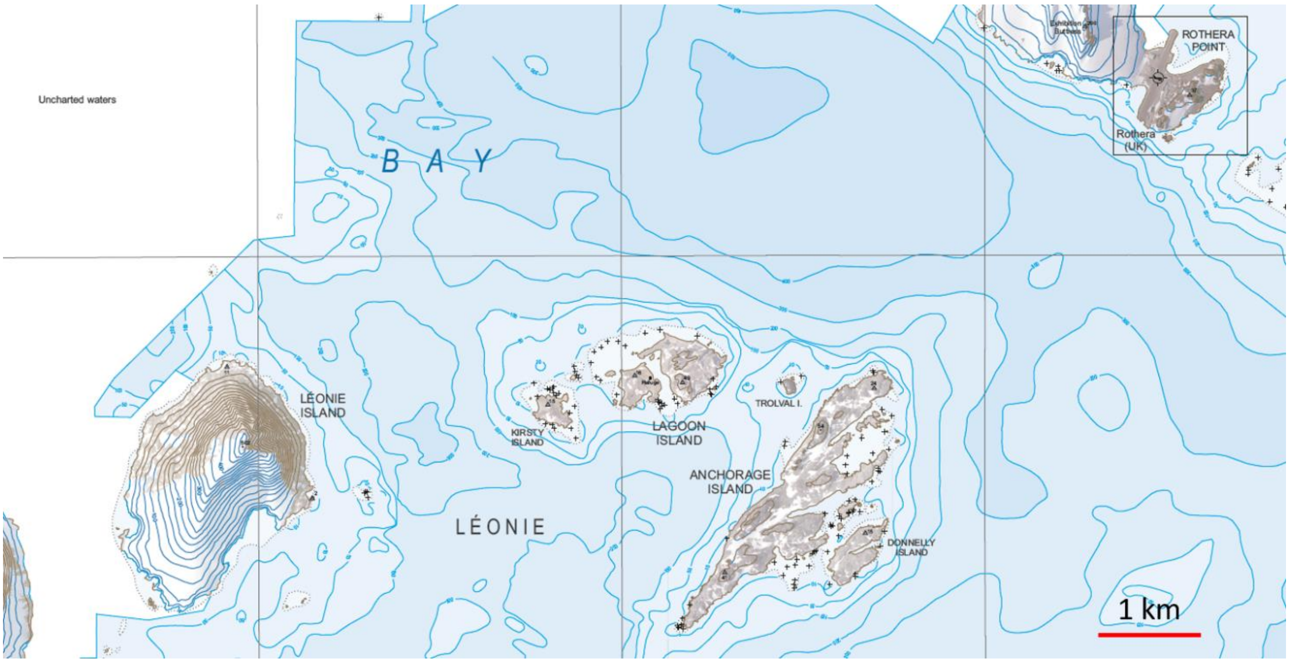

Table S2 Supporting Information Table S2: FT-IR metabolic fingerprinting of snow algae communities. Absorption bands and wavenumber (cm-1) ranges of main functional groups and the associated wavenumbers that are positively associated with either green or red snow algae communities collected from four locations in maritime Antarctica in austral summer 2014/15.

Functional groups	Wavenumber range	Green community wavenumber range	Red community wavenumber range
Lipid methyl/methylene bands v_{as} CH_3, v_s CH_3, v_{as} CH_2 v_s CH_2	3050-2800		2856-2850
Lipid ester bands V_s > C=O	1850-1700		1756-1732
Protein bands (amide I) v_s C=O	1700-1600	1664-1589	
Protein bands (amide II) δ N-H, v_s C-N	1600-1500	1581-1489	
Polysaccharide bands v_s CO-O-C	1200-930		1094-1002; 1144-1141

v = stretching, δ = bending, *a* = symmetric, *as* = asymmetric. Band classifications based on Benning *et al*., 2004, Mayers *et al*., 2013, Lutz *et al*., 2015.

Table S3 Supporting Information Table S3: Fatty acid composition of snow algae. Mean concentrations of total fatty acyl methyl esters (FAMEs, expressed as mg g-1 dry cell mass) of green and red snow algal communities collected from four locations in the maritime Antarctic (Rothera Point, Anchorage Island, Léonie Island and Lagoon Island) during austral summer (Jan–Feb) 2015. Values in bold are the most abundant FAMEs detected.

	Rothera Point		Anchorage Island		Léonie Island		Lagoon Island		**All locations**		General trend of difference between all green and red communities
Fatty acid<br>ınsaturation positions)	Green<br>(SD, *n* = 2)	Red<br>(SD, *n* = 1)	Green<br>(SD, *n* = 2)	Red<br>(SD, *n* = 5)	Green<br>(SD, *n* = 1)	Red<br>(SD, *n* = 2)	Green<br>(SD, *n* = 3)	Red<br>(SD, *n* = 2)	**Green**<br>**(SD, *n* = 8)**	**Red**<br>**(SD, *n* = 10)**	
.4:0	1.3 (1.8)	0.7 (0.0)	0.2 (0.1)	0.9 (0.7)	0.5 (0.0)	0.3 (0.4)	1.1 (1.6)	1.3 (1.3)	0.9 (1.2)	0.8 (0.7)	↔
**.6:0**	**4.8 (5.6)**	**6.7 (0.0)**	**2.8 (2.5)**	**9.4 (5.6)**	**4.7 (0.0)**	**7.3 (0.1)**	**21.0 (19.9)**	**22.1 (0.1)**	**10.4 (14.0)**	**11.3 (6.9)**	↑
.6:1 (9)	1.2 (1.5)	0.0 (0.0)	0.4 (0.2)	0.5 (0.1)	3.0 (0.0)	0.1 (0.1)	1.3 (1.2)	0.2 (0.0)	1.3 (1.2)	0.3 (0.2)	↓
.6:1 (7)	0.1 (0.1)	0.0 (0.0)	0.1 (0.1)	0.1 (0.1)	0.3 (0.0)	0.0 (0.1)	0.6 (0.7)	0.1 (0.0)	0.3 (0.5)	0.1 (0.1)	↓
.6:2 (7,10)	0.3 (0.3)	0.0 (0.0)	0.1 (0.1)	0.2 (0.1)	0.3 (0.0)	0.1 (0.1)	0.6 (0.8)	0.1 (0.1)	0.4 (0.5)	0.1 (0.1)	↓
.6:3 (7,10,13)	1.5 (1.8)	0.0 (0.0)	0.6 (0.4)	0.7 (0.4)	2.0 (0.0)	0.2 (0.2)	2.1 (1.9)	0.5 (0.4)	1.6 (1.4)	0.5 (0.4)	↓
.6:4 (4,7,10,13)	3.3 (3.7)	0.0 (0.0)	1.4 (1.2)	1.6 (0.6)	6.6 (0.0)	0.2 (0.3)	4.5 (4.3)	0.9 (1.0)	3.7 (3.2)	1.0 (0.8)	↓
.8:0	0.8 (1.0)	3.6 (0.0)	0.6 (0.7)	2.0 (1.2)	0.6 (0.0)	1.4 (1.9)	6.7 (8.8)	5.2 (0.8)	3.0 (5.7)	2.7 (1.8)	↔
**.8:1 (11)**	**14.1 (16.2)**	**0.5** (0.0)	**7.4 (6.2)**	**22.7 (17.0)**	**18.4** (0.0)	**8.1 (11.3)**	**46.0 (29.0)**	**94.0 (44.3)**	**24.9 (24.5)**	**31.8 (38.8)**	↑
.8:1 (10)	0.1 (0.1)	0.1 (0.0)	0.1 (0.1)	0.2 (0.1)	0.1 (0.0)	0.1 (0.0)	0.6 (0.3)	1.0 (1.0)	0.3 (0.3)	0.3 (0.5)	↔
.8:1 (9)	0.6 (0.7)	0.8 (0.0)	0.3 (0.3)	0.8 (0.4)	0.6 (0.0)	0.6 (0.7)	3.5 (2.4)	11.4 (9.5)	1.9 (2.0)	2.9 (5.5)	↑
.8:2 (9,12)	2.0 (2.5)	0.0 (0.0)	0.6 (0.4)	0.9 (0.6)	2.3 (0.0)	0.2 (0.3)	2.9 (1.4)	1.1 (0.2)	2.0 (1.5)	0.7 (0.6)	↓
.8:3 (6,9,12)	0.1 (0.2)	0.0 (0.0)	0.0 (0.0)	0.1 (0.0)	0.1 (0.0)	0.0 (0.1)	0.3 (0.3)	0.0 (0.0)	0.2 (0.2)	0.0 (0.0)	↓
.8:4 (6,9,12,15)	2.4 (3.2)	0.0 (0.0)	0.5 (0.3)	0.6 (0.3)	2.0 (0.0)	0.1 (0.1)	1.2 (1.1)	0.4 (0.1)	1.4 (1.5)	0.4 (0.3)	↓
!0:0	0.0 (0.1)	0.1 (0.0)	0.0 (0.0)	0.1 (0.0)	0.0 (0.0)	0.1 (0.1)	0.3 (0.2)	0.5 (0.0)	0.1 (0.2)	0.2 (0.2)	↔
!2:6 (4,7,10,13,16,19)	0.5 (0.7)	0.0 (0.0)	0.0 (0.0)	0.0 (0.0)	0.0 (0.0)	0.0 (0.0)	0.0 (0.0)	0.0 (0.0)	0.1 (0.4)	0.0 (0.0)	↔
**Sum FAMEs**	**33.2 (39.6)**	**12.4 (0.0)**	**15.3 (12.7)**	**40.7 (24.0)**	**41.6 (0.0)**	**19.0 (15.8)**	**92.8 (72.1)**	**138.9 (50.8)**	**52.1 (54.3)**	**53.1 (52.3)**	↔

Table S4 Supporting Information Table S4: Score contribution of putatively identified metabolites associated with either green (n = 11) or red (n = 14) algae communities. Score values are ranked in descending order of importance and are positive if they contribute towards PCA loading plots for the green snow algae communities and negative if they contribute towards the red snow algae communities.

Green community		Red community	
**Putative metabolite name**	**Score contribution value (PC1-PC3) of metabolite (Descending order of importance for green samples)**	**Putative metabolite name**	**Score contribution value (PC1-PC3) of metabolite (Descending order of importance for red samples)**
Asparagine	2.49	Mannitol	-0.72
Succinic acid	2.42	Heptadecanoic acid	-0.54
Valine	2.18	Xylitol	-0.52
Aminoadipic acid	2.02	Dimethylheptanoic acid	-0.49
Lysine	1.90	Phenylethanolamine	-0.49
Glutamic acid	1.84	Galactopyranoside	-0.39
Inositol-2-phosphate	1.70	Glycerol	-0.33
Calystegine	1.63	Ribo-Hexitol	-0.31
Lactic acid	1.60	Gulonic acid	-0.30
Leucine	1.56	Cystathionine	-0.29
Alanine	1.53	Isoleucine	-0.27
Melibiose	1.51	Octadecenoic acid	-0.26
Galactosylglycerol	1.38	Fructose	-0.26
Glyceraldehyde-3-phosphate	1.25	Sucrose	-0.26
Inosine	1.25	Dimethylbenzoic acid	-0.25
Malic acid	0.80	Galacturonide	-0.23
Threonine	0.76	Glucofuranose	-0.23
Putrescine	0.71	Monolinoleoylglycerol	-0.22
Trehalose	0.33	Carbamic acid	-0.22
Cyclooctanecarboxylic acid	0.25	Galactonolactone	-0.22
Cysteine	0.25	Glucosamine	-0.22
Erythro-Pentitol	0.25	Hexadecanenitrile	-0.21
Glucuronic acid	0.25	Lactose	-0.21
Hydroxybutyric acid	0.25	Erythritol	-0.21
Nitrobarbiturate	0.25	Glucose	-0.21
Tyrosine	0.25	Cellobiose	-0.19
Uracil	0.25	Galactopyranose	-0.19
Uridine	0.25	Sorbose	-0.19
Mannopyranoside	0.25	Octadecatrienoic acid	-0.17
Hypoxanthine	0.25	Erythrofuranose	-0.16

Inositol	0.20	Xylose	-0.16
Hexitol	0.18	Tetradecanoic acid	-0.16
Galactose-6-phosphate	0.18	Allantoin	-0.16
Phosphoric acid	0.17	Guanine	-0.15
Fumaric acid	0.17	Oleanitrile	-0.15
Indole	0.16	Tyramine	-0.14
Ketogluconic acid	0.15	Aminobutyric acid	-0.14
Psicofuranose	0.14	Gulono-1,4-lactone	-0.13
Butenedioic acid	0.13	Mercaptosuccinic acid	-0.13
Carbamate	0.13	Glucopyranosiduronic acid	-0.13
Mannose-6-phosphate	0.13	Mannopyranosyl	-0.13
Ribose	0.13	Dehydroascorbic acid	-0.11
Adenosine	0.13	Octanoic acid	-0.11
Hexanoic acid	0.11	Maltose	-0.09
Tagatofuranose	0.10	Fructofuranose	-0.08
Glyceric acid	0.10	Propanoic acid	-0.08
Gluconic acid	0.09	Ascorbic acid	-0.08
Eicosatetraynoic acid	0.09	Dodecanoic acid	-0.06
Pyroglutamic acid	0.09	Octanoate	-0.06
Decanoic acid	0.09	Fucitol	-0.05
Pentadecane	0.08	Hexadecanoic acid	-0.05
Butanoic acid	0.06	Octadecanoic acid	-0.05
Tricosadiynoic acid	0.06	Acetylglutamic acid	-0.03
Sorbitol	0.06	Proline	-0.02
Serine	0.05	Tagatose	-0.02
Docosahexaenoic acid	0.05	Urea	-0.02
Threonic acid	0.04	Uric acid	-0.02
Glucopyranoside	0.03	Erythrose	-0.02
Galactose	0.03	Threose	-0.01
Mannose	0.02	Methionine	-0.01
Arabinopyranose	0.02	Pentitol	-0.01

Table S5 Supporting Information Table S5. Metabolite pathways of the putatively identified metabolites associated with either green or red snow algae communities. Pathways are ranked in descending order of importance based on the total number of metabolites within a pathway and the number of identified metabolites within the pathway that were identified as contributing towards separating green and red samples within PCA score scatter plots. In silico analysis was performed with MetaboAnalyst v4 Pathway Analysis Tool using the Arabidopsis thaliana metabolic pathway library. Hits of 1 are omitted.

**Green community**		
**Pathway name (ranked on descending order of metabolite hits from green samples)**	**Total number of metabolites in pathway**	**Number of identified metabolite hits matching those in pathway**
Aminoacyl-tRNA biosynthesis	67	16
Arginine and proline metabolism	38	9
Alanine, aspartate and glutamate metabolism	22	7
Nitrogen metabolism	15	4
Glutathione metabolism	26	5
Galactose metabolism	26	5
Cyanoamino acid metabolism	11	3
Fructose and mannose metabolism	16	3
Glycine, serine and threonine metabolism	30	4
Tyrosine metabolism	18	3
Purine metabolism	61	6
Citrate cycle (TCA cycle)	20	3
Cysteine and methionine metabolism	34	4
Lysine biosynthesis	10	2
Methane metabolism	11	2
Sulfur metabolism	12	2
Valine, leucine and isoleucine biosynthesis	26	3
Ascorbate and aldarate metabolism	15	2
Glyoxylate and dicarboxylate metabolism	17	2
Pentose phosphate pathway	18	2
Butanoate metabolism	18	2
Phenylalanine, tyrosine and tryptophan biosynthesis	21	2
Carbon fixation in photosynthetic organisms	21	2
Pyrimidine metabolism	38	3
Inositol phosphate metabolism	24	2

**Red Community**		
**Pathway name (ranked on descending order of metabolite hits from red samples)**	**Total number of metabolites in pathway**	**Number of identified metabolite hits matching those in pathway**
Galactose metabolism	26	4
Starch and sucrose metabolism	30	4
Fatty acid biosynthesis	49	5
Ascorbate and aldarate metabolism	15	2
Arginine and proline metabolism	38	3
Cysteine and methionine metabolism	34	2
Purine metabolism	61	3
Aminoacyl-tRNA biosynthesis	67	3
Amino sugar and nucleotide sugar metabolism	41	2
Biosynthesis of unsaturated fatty acids	42	2
Glucosinolate biosynthesis	54	2

Glucosinolate biosynthesis	54	3
Valine, leucine and isoleucine degradation	34	2
Amino sugar and nucleotide sugar metabolism	41	2

Table S6 Supporting Information Table S6. Percentage contributions and number of taxonomic assignments for Level 2 (Kingdom/Phylum) 99% aligned OTUs for 16S rRNA gene and ITS1 (18S) sequences in green and red snow algae communities from Ryder Bay, Antarctica. Percentage contribution values are the mean relative abundance of the taxon in percentage of total sequences with more than 0.5% abundance. Low abundance OTU values are the sum of the percentages for taxa identified below 0.5% contribution. All values are means of n = 5 (green communities) and n = 6 (red communities) +/- standard deviation and are ranked in descending order of green community values. SAR = “stramenopiles, alveolata, rhizaria”. None of the values were statistically different between communities at this taxonomy level.

16S rRNA gene Level 2 (Phylum) taxonomy assignment				
	Percent contribution		Number of OTU reads	
	Green	Red	Green	Red
Bacteroidetes	62.1 (17.2)	56.5 (11.6)	52566 (30515)	41763 (21320)
Proteobacteria	22.0 (13.1)	27.1 (12.0)	17645 (11782)	18226 (7842)
Unknown Bacteria	6.1 (4.3)	9.6 (4.9)	4429 (2806)	6545 (3250)
Cyanobacteria	4.2 (4.8)	3.0 (2.1)	2855 (3192)	1957 (1237)
Archaeplastida	2.1 (1.6)	0.8 (0.4)	1918 (2286)	539 (291)
Actinobacteria	1.9 (0.9)	1.5 (0.6)	1700 (1528)	1045 (507)
Unclassified	0.6 (0.3)	0.5 (0.2)	522 (489)	358 (158)
Low abundant OTUs	0.9 (0.1)	1.1 (0.1)	713 (61)	755 (60)
Shannon’s Diversity Index			1.15	1.18

ITS1 (18S) Level 2 (Phylum) taxonomy assignment				
	Percent contribution		Number of OTU reads	
	Green	Red	Green	Red
SAR	49.8 (37.1)	37.2 (43.0)	444 (477)	825 (1732)
Opisthokonta	39.9 (39.0)	43.6 (41.6)	362 (460)	85 (97)
Excavata	5.6 (12.5)	0.2 (0.4)	10 (21.9)	4 (6)
Archaeplastida	4.0 (4.7)	16.4 (22.3)	13 (10)	25 (29)
Unclassified	0.7 (1.0)	2.6 (3.8)	7 (13)	56 (134)
Shannon’s Diversity Index			0.86	0.64

Table S7 Supporting Information Table S7. Percentage contributions and number of taxonomic assignments for Level 3 (Order, Class) 99% aligned OTUs for 16S rRNA gene and ITS1 (18S) sequences in green and red snow algae communities from Ryder Bay, Antarctica. Percentage contribution values are the mean relative abundances of the taxa in percentage of total sequences with more than 0.5% abundance. Low abundance OTU values are the sum of the percentages for taxa identified below 0.5% contribution. All values are means of n = 5 (green communities) and n = 6 (red communities) +/- standard deviation and are ranked in descending order of green community values. * = P < 0.05 (between green and red communities).

16S rRNA gene Level 3-4 (eg. Class) taxonomy assignment					18S ITS1 Level 2 (eg. Class) taxonomy assignment				
	Percent contribution		Number of OTU reads			Percent contribution		Number of OTU reads	
	Green	Red	Green	Red		Green	Red	Green	Red
Flavobacteria	33.1 (21.5)	22.5 (8.6)	29746 (25541)	15185 (5467)	Unknown Alveolata (SAR)	49.7 (36.9)	36.8 (42.4)	443 (477)	804 (1682)
Sphingobacteria	21.3 (5.4)	11.5 (8.9) *	16805 (6433)	8230 (6282) *	Tremellaceae (Fungi)	26.5 (38.6)	8.4 (11.9)	145 (218)	13 (20)
Betaproteobacteria	17.2 (11.1)	17.8 (10.7)	14094 (10725)	12092 (7482)	Rhizophydiales (Fungi)	12.8 (28.0)	12.7 (31.2)	211 (463)	15 (36)
Cytophagia	7.5 (5.1)	22.4 (21.2)	5885 (3696)	18287 (19559)	Tetramitia (Excavata)	5.6 (12.5)	0.0 (0.0)	10 (22)	0 (0)
Unknown Bacteria	6.1 (4.3)	9.6 (4.9)	4429 (2806)	6545 (3250)	Chlorophyceae	3.4 (5.0)	15.5 (22.7)	10 (12)	22 (26)
Cyanobacteria Chloroplast	4.1 (4.9)	2.9 (2.0)	2762 (3218)	1846 (1189)	Unclassified	0.7 (1.0)	2.6 (3.8)	7 (13)	56 (134)
Gammaproteobacteria	3.7 (2.2)	8.4 (13.0)	2682 (1396)	5588 (9079)	Trebouxiophyceae (Chlorophyta)	0.6 (1.3)	0.0 (0.0)	3 (8)	0 (0)
Chloroplastida	2.1 (1.6)	0.8 (0.4)	1918 (2286)	539 (291)	Dothideomycetes (Fungi)	0.1 (0.3)	7.4 (17.0)	1 (2)	20 (48)
Actinobacteria	1.9 (0.9)	1.5 (0.6)	1674 (1542)	1033 (496)	Unknown Fungi	0.0 (0.0)	9.4 (22.8)	0 (0)	29 (63)
Alphaproteobacteria	0.8 (0.4)	0.8 (0.5)	608 (323)	512 (358)	Dikarya (Fungi)	0.0 (0.0)	5.7 (13.6)	0 (0)	9 (14)
Unclassified	0.6 (0.3)	0.5 (0.2)	522 (489)	358 (158)	Unknown Chlorophyta	0.0 (0.0)	0.9 (2.1)	0 (0)	3 (4)
Low abundance OTUs	1.6 (0.1)	1.4 (0.1)	1222 (46)	975 (43)	Low abundance OTUs	0.6 (0.2)	0.6 (0.1)	6 (2)	24 (6)
Shannon’s Diversity Index			1.9	2.0	Shannon’s Diversity Index			1.20	0.89

Table S8 Supporting Information Table S8. Percentage contributions and numbers of taxonomic assignments for Level 6 (Family, Genus) 99% aligned OTUs for 16S rRNA gene and ITS1 (18S) sequences in green and red snow algae communities from Ryder Bay, Antarctica. Percentage contribution values are the mean relative abundance of the taxa in percentage of total sequences with more than 0.5% abundance. Low abundance OTU values are the sum of the percentages for taxa identified below 0.5% contribution. All values are means of n = 5 (green community sites) and n = 6 (red community sites) +/- standard deviation and are ranked in descending order of green community values. * = P < 0.05; ** = P < 0.01 (between green and red communities).

16S rRNA gene Level 6 (e.g. genus) taxonomy assignment					18S ITS1 Level 6 (e.g. genus) taxonomy assignment				
	Percent contribution		Number of OTU reads			Percent contribution		Number of OTU reads	
	Green	Red	Green	Red		Green	Red	Green	Red
Flavobacterium	28.4 (23.6)	10.2 (8.5)	26739 (27045)	6139 (3681)	Unknown_Alveolata (SAR)	49.7 (36.9)	36.8 (42.4)	443 (477)	804 (1682)
Pedobacter	14.9 (8.2)	9.2 (7.2)	12338 (7181)	6667 (5286)	Unknown *Cryptococcus* (Fungi)	26.5 (38.6)	8.4 (11.9)	145 (218)	13 (20)
Hymenobacter	6.4 (5.9)	22.2 (21.3)	4783 (4158)	18126 (19626)	Unknown Rhizophydiales (Fungi)	12.8 (28.0)	12.7 (31.2)	211 (463)	15 (36)
Unknown Bacteria	6.1 (4.3)	9.6 (4.9)	4429 (2806)	6545 (3250)	*Tetramitis* (Excavata)	5.6 (12.5)	0.0 (0.0)	10 (22)	0 (0)
Massilia	5.8 (9.5)	1.6 (2.8)	3278 (4996)	904 (1217)	Uncultured *Chloromonas* (Chlorophyta)	2.5 (5.0)	6.0 (11.1)	5 (8)	5 (6)
Unknown Sphingobacteriaceae	5.5 (6.7)	1.6 (1.5)	3758 (3923)	1127 (1043)	*Chlamydomonas* (Chlorophyta)	0.9 (2.1)	0.0 (0.0)	4 (9)	0 (0)
Polaromonas	5.1 (3.3)	6.2 (3.8)	4801 (5356)	4526 (3514)	Unclassified	0.7 (1.0)	2.6 (3.8)	7 (13)	56 (134)
Unknown Comamonadaceae	5.0 (3.2)	9.1 (5.7)	4966 (5319)	6120 (3863)	*Chlorella* (Chlorophyta)	0.6 (1.3)	0.0 (0.0)	3 (8)	0 (0)
Unknown Cyanobacteria	4.1 (4.9)	2.9 (2.0)	2761 (3218)	1846 (1189)	Unknown Fungi	0.0 (0.0)	9.4 (22.8)	0 (0)	29 (63)
Chryseobacterium	4.0 (4.1)	11.4 (2.8) **	2576 (5290)	8377 (4666) *	Unknown Chlorophyceae	0.0 (0.0)	9.1 (21.6)	0 (0)	16 (26)
Unknown Chlorophyceae	1.7 (1.5)	0.4 (0.2)	1606 (1989)	311 (190)	Unknown Pleosporales (Fungi)	0.0 (0.0)	7.0 (17.1)	0 (0)	20 (48)

Rhodanobacter	1.7 (1.7)	6.8 (13.3)	1068 (1083)	4522 (9259)
Unknown Microbacteriaceae	1.5 (0.9)	1.0 (0.4)	1390 (1458)	757 (352)
Unknown Oxalobacteraceae	0.8 (0.5)	0.7 (0.9)	498 (151)	392 (419)
Arcicella	0.7 (1.2)	0.0 (0.0)	692 (1240)	8 (18)
Pseudomonas	0.6 (0.7)	0.3 (0.5)	448 (426)	128 (192)
Unclassified	0.6 (0.3)	0.5 (0.2)	522 (489)	358 (158)
Marinobacter	0.3 (0.4)	1.0 (1.8)	260 (307)	675 (1081)
Low abundance OTUs	6.7 (0.1)	5.3 (0.1)	5433 (48)	3662 (37)
Shannon's Diversity Index			2.61	2.58

Unknown Basidiomycota (Fungi)	0.0 (0.0)	5.7 (13.6)	0 (0)	9 (14)
Unknown Chlorophyta	0.0 (0.0)	0.9 (2.1)	0 (0)	3 (4)
Low abundance OTUs	0.7 (0.1)	1.5 (0.2)	7 (2)	25 (6)
Shannon's Diversity Index			1.21	0.91

Table S9 Supporting Information Table S9: Targeted genomic identification of snow algae. Top three sequence alignments of forward BLAST searches on 18S rRNA gene sequences (from “SA Forward” primer, GenBank accession numbers MK330877 and MK330879) from axenic green snow algae collected from Lagoon Island, Antarctic Peninsula during austral summer 2015. Sequences were aligned against datasets containing either all species, Chloromonas nivalis only or Chlamydomonas nivalis only using BLAST (National Center for Biotechnology Information, NCBI). All alignments have an E value of 0.0 with a query coverage of 98%.

**NCBI data set searched**	**Identity match**	**NCBI accession**	**Location origin of sequenced species**	**NCBI species description**
All species	99%	KU886306.1	Arctic freshwater	*Chloromonas* sp. KNF0032
All species	99%	HQ404890.1	Antarctica	*Chloromonas* sp. CCCryo273-06 strain CCCryo 273-06
All species	98%	EF106784.1	Uni of Nevada	*Chlamydomonas* sp. CCMP681
Chloromonas nivalis	92%	AF514409.1	Svalbard snow	*Chloromonas nivalis* strain CCCryo 005-99
Chloromonas nivalis	92%	GU117576.1	Austria red snow	*Chloromonas nivalis*
Chloromonas nivalis	92%	AF514406.1	Svalbard snow	*Chloromonas nivalis* strain CCCryo 047-99
Chalmydomonas nivalis	92%	JQ790560.1	Svalbard red snow	*Chlamydomonas nivalis* strain CCCryo RS_0015-2010
Chalmydomonas nivalis	92%	JQ790558.1	Svalbard red snow	*Chlamydomonas nivalis* strain CCCryo RS_0005-2004
Chalmydomonas nivalis	92%	JQ790557.1	Svalbard red snow	*Chlamydomonas nivalis* strain CCCryo RS_0006-2004

Methods S1 R script. Raw read metabarcoding data R script in vegan. Zip R script files are attached separately.

References:

Benning LG, Phoenix VR, Yee N, Tobin MJ. 2004. Molecular characterization of cyanobacterial silicification using synchrotron infrared micro-spectroscopy. *Geochimica et Cosmochimica Acta* **68**: 729–741.

Lutz S, Anesio AM, Field K, Benning LG. 2015. Integrated 'Omics', Targeted metabolite and single-cell analyses of Arctic snow algae functionality and adaptability. *Frontiers in Microbiology* **6**: 1323 doi:10.3389/fmicb.2015.01323

Mayers JJ, Flynn KJ, Shields RJ. 2013. Rapid determination of bulk microalgal biochemical composition by Fourier Transform Infrared Spectroscopy. *Bioresource* Technology **148**: 215-220.

Paliy O, Shankar V. 2016. Application of multivariate statistical techniques in microbial ecology. *Molecular Ecology* **25**: 1032–1057.
